# From gut to joint: the protective impact of *Eubacterium rectale* on rheumatoid arthritis

**DOI:** 10.3389/fimmu.2025.1607804

**Published:** 2025-07-25

**Authors:** Xiaoyang Liu, Ruihe Wu, Yuxin Fan, Kaili Qin, Baochen Li, Xiaofeng Li, Chong Gao, Caihong Wang

**Affiliations:** ^1^ Department of Rheumatology, The Second Hospital of Shanxi Medical University, Taiyuan, Shanxi, China; ^2^ Shanxi Key Laboratory of Rheumatism Immune Microecology, Taiyuan, Shanxi, China; ^3^ Shanxi Precision Medical Engineering Research Center for Rheumatology, Taiyuan, Shanxi, China; ^4^ Department of Pathology, Brigham and Women’s Hospital/Children’s Hospital Boston, Joint Program in Transfusion Medicine, Harvard Medical School, Boston, MA, United States

**Keywords:** rheumatoid arthritis, *Eubacterium rectale*, intestinal flora, immune regulation, autoimmune disease

## Abstract

**Background:**

Rheumatoid arthritis (RA) is a chronic autoimmune disease with a complex and diverse etiology. The onset of RA is closely associated with intestinal flora, which is essential for immune regulation.

**Methods:**

Fecal samples of 22 healthy controls and 38 patients with newly diagnosed RA were used for performing 16S rRNA sequencing, microbiota diversity assessment, and functional enrichment analysis. Through integrative analysis of random forest feature selection and bidirectional Mendelian randomization (MR), *Eubacterium rectale* was prioritized as a key bacterial candidate associated with RA. Furthermore, *E. rectale* was used to treat the arthritis model mice by gavage treatment, and we evaluated joint inflammation and immune cell profile in mice. Finally, untargeted metabolomics was used to evaluate the changes in serum and fecal metabolites in the arthritis mouse model before and after *E. rectale* intervention.

**Results:**

The beta diversity of the intestinal flora exhibited significant differences between RA patients and healthy controls (HC). Functional enrichment analysis revealed that RA patients’ intestinal microbiota functions were enriched in pathways like genetic information processing and material metabolism. Further random forest model revealed *E. rectale, Bacteroides*, etc., and twelve genera with characteristic significance in RA patients. According to further MR analysis, *Anaerostipes* and *E. rectale* had a protective effect on RA, and reverse MR analysis showed no evidence of a causal relationship between these groups and RA. *In vivo* experiments showed that after the administration of *E. rectale*, the joint inflammation of the mice was relatively slight, the bone destruction and bone density of the joints improved, the proportion of Treg and follicular regulatory T cells (Tfr) cells increased, and the proportion of follicular helper T cells (Tfh) cells decreased. Metabolomic analysis revealed significant changes in both serum and fecal metabolites in mice with collagen-induced arthritis (CIA) compared with healthy controls. The changes in metabolites such as butyric acid were reversed after treatment with *E. rectal*.

**Conclusion:**

The study demonstrates that *E. rectale* has a protective effect on RA. *E. rectale* significantly attenuates joint inflammation in mouse models by may regulating the expression level of butyrate, ameliorating the Treg and Tfr/Tfh immune imbalance status, and re-establishing the immune tolerance. These findings serve as valuable references for future studies on the pathogenesis of RA and the development of new therapeutic approaches.

## 1 Introduction

Rheumatoid arthritis (RA) is a chronic autoimmune disease that causes inflammation in the joints. It is caused by a breakdown in immunological tolerance. This leads to cartilage damage, bone erosion, and disability, often affecting multiple systems and organs beyond the joints. The range of RA prevalence is 0.4% to 1.3% (1). In recent years, disease-modifying antirheumatic drugs (DMARDs) and biologics have been widely used, greatly decreasing RA-related morbidity, disability, and morbidity while also improving patients’ quality of life (2). However, RA is a highly heterogeneous disease, which has led to a large gap in the early treatment and prevention of RA, mainly manifested in the limited means of identifying RA in the preclinical stage and the fact that treatment is ineffective for many patients, fail to achieve immune homeostasis remodeling or have no drug remission (3, 4). Addressing these unresolved issues and exploring disease heterogeneity requires a deeper understanding of the pathogenesis of RA.

The development and imbalance of immune tolerance are closely related to changes in the human microbiome. Mucosal flora dysbiosis, or changes in the microbial balance in the mouth, lungs, and intestines, has been shown in previous studies to be a significant contributor to the development of RA (5). Particularly, it has been demonstrated that the gut flora has an immunomodulatory influence and is associated with the development of RA. The reason is that the influence of intestinal flora metabolites on the immune system is the basis of the gut-joint axis. Manipulating the intestinal microbiome provides an alternative strategy for alleviating RA (6, 7). Previous studies have confirmed that microbes such as *Clostridium butyricum*, *Bacteroides fragilis*, etc. are involved in the occurrence of RA and have been applied in clinical treatment, which gives us great confidence to further explore the abundance and diversity of intestinal flora. In view of the complexity and diversity of intestinal flora, existing strategies for the treatment of intestinal flora are not suitable for all patients, and there are still many potentially key microbiotas that have not been identified.

**Figure 1:**
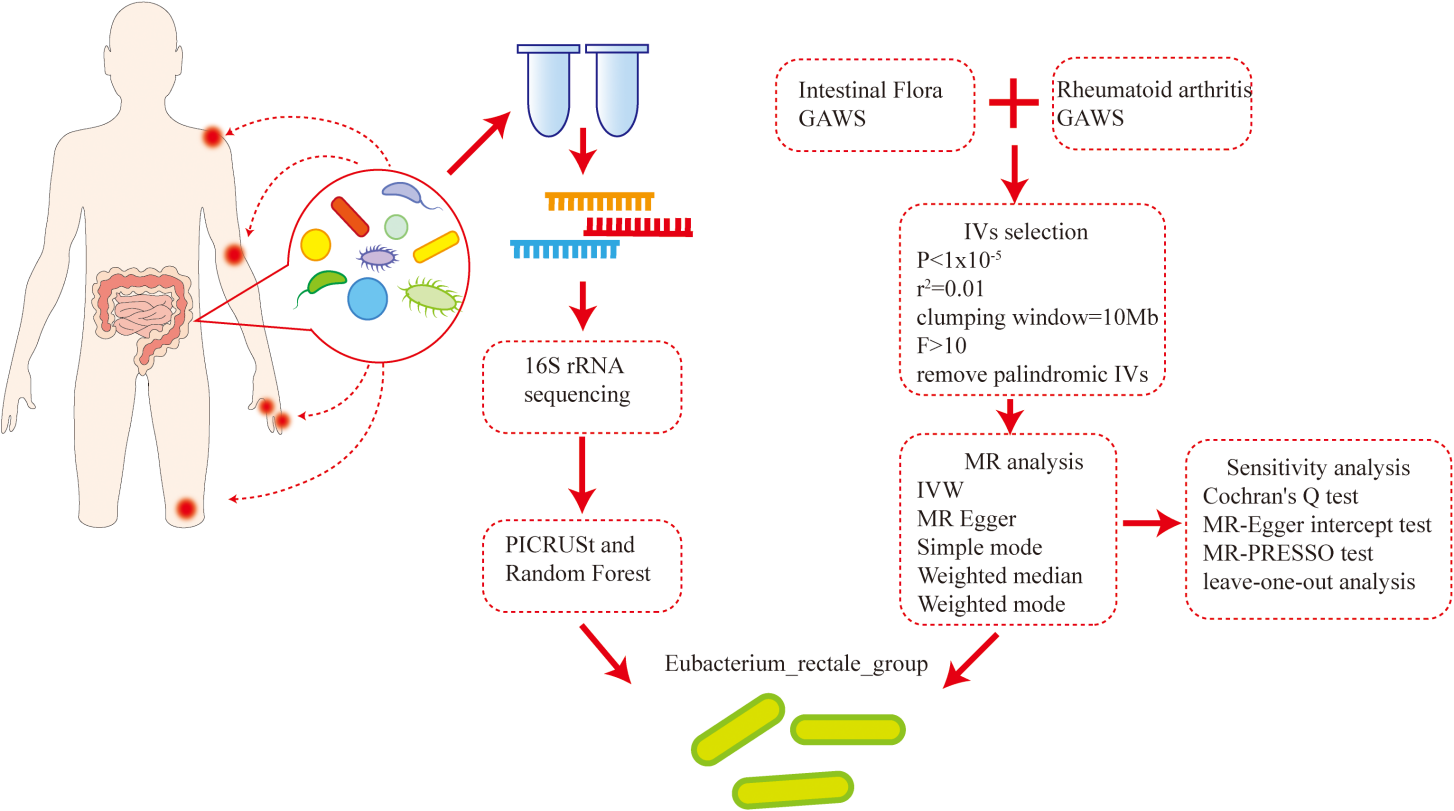
Flowchart illustrating the main hypotheses and methods of the current study.

This study conducted 16S rRNA sequencing on the feces of 38 newly diagnosed RA patients and 22 healthy individuals, elucidating the differences in intestinal flora composition between the two groups. Then, utilizing intestinal microbial flora and RA-related GWAS data, bidirectional two-sample Mendelian randomization (MR) analysis was performed (8, 9) to investigate any possible association between alterations in intestinal microbial flora and RA. By conjoint analysis, *Eubacterium rectale* was identified as a specific bacterial species demonstrating a significant association with the development of RA. Then, we experimentally verified our findings by using a model of collagen-induced arthritis (CIA) mice induced by bovine type II collagen. The results confirmed that *E. rectale* can alleviate joint inflammation in CIA mice and regulate immune imbalance in the mice. This discovery provides a new clear target for probiotic immunotherapy of RA, potentially enhancing treatment effectiveness and improving patient prognosis (**Figure 1**).

## 2 Materials and methods

### 2.1 Study population and data

In this study, individuals with newly diagnosed RA were recruited at Shanxi Medical University’s Second Hospital between June 2023 and June 2024. For at least 3 months prior to the experimental initiation, participants had to abstain from the use of steroids, biologics, or DMARDs, and meet the diagnostic and classification criteria of the 2010 ACR/EULAR RA. The healthy control group (HC) was simultaneously chosen from healthy volunteers who had no prior history of autoimmune diseases or abnormal clinical indicators.

The strict exclusion criteria included a history of gastrointestinal surgery, use of antibiotics or microecological drugs within the previous 8 weeks, malignant tumors, severe infections, severe cardiovascular diseases, inflammatory bowel disease, and other conditions that could significantly affect the intestinal microbiota and its metabolites for both new-onset RA patients and healthy individuals. Finally, 22 healthy controls and 38 new-onset RA patients were selected to participate in this study after screening. The study was conducted in compliance with the Declaration of Helsinki and received ethical approval from Shanxi Medical University’s Second Hospital (approval number: 2021 YX No.250). Written informed permission was acquired for each subject. Stool samples from both newly diagnosed RA patients and healthy controls were obtained during the study and stored at -80°C for 16S rRNA sequencing.

### 2.2 Fecal DNA extraction and 16S rRNA sequencing and data analysis

Total DNA was extracted from 200 mg of pretreated stool samples following the comprehensive instructions provided by the E.Z.N.A. Fecal DNA Kit (D4015, Omega, Inc., USA) (10). We next focused on the highly variable region (V3-V4) of the 16S rRNA gene and performed targeted polymerase chain reaction (PCR) amplification. In all PCR reactions, which were carried out in a 30 μL reaction volume, the forward primer (341F: 5’-CCTACGGGNGGCWGCAG-3’) and the reverse primer (805R: 5’-GACTACHVGGGTATCTAATCC-3’) were utilized. The initial step in the thermal cycling process was denaturation at 98°C for 1 min, 30 cycles of annealing at 50°C for 30 s, extension at 72°C for 60 s, and denaturation for 10 s at 98°C, followed by a final extension at 72°C for 5 min. A detailed analysis of the amplified products was conducted using 2% agarose gel electrophoresis. After confirming their specificity and integrity, we purified and accurately quantified the PCR products. Next, we constructed a hybridization library and denatured it to single-stranded DNA.

We transferred the generated library onto the Illumina sequencing platform for high-throughput sequencing using a double-end sequencing strategy (2 x 250 bp). The paired-end reads obtained by sequencing were efficiently merged by FLASH software (version v1.2.8) (11) and then strictly screened by the FQTrim software (version v0.94) (12).

The raw data were finely processed by an in-house processing pipeline customized based on Mothurv version 1.31.2 (13). Low-quality sequences were eliminated if their average quality score was less than the 20 bp threshold, and integrated all high-quality sequence reads longer than 250 bp. Subsequently, the sequences were clustered using USEARCH software (v10.0.240) (14) and divided into non-singleton amplicon sequence variants (ASVs) using the non-clustered denoising method. On this basis, we employed vsearch (v2.15.2) (15) to generate feature tables. Subsequently, taxonomic annotation of bacterial and archaeal 16S rRNA genes was performed using the SILVA 123 database (16). We used α diversity metrics (Chao1, Shannon, Ace), β diversity metrics (PcoA, NMDS, ANOSIM) to evaluate the differences between samples.

The 16S rRNA feature sequences underwent reference-based alignment to construct a phylogenetic tree. Employing the castor R package’s hidden state prediction algorithm, gene family copy numbers were inferred by identifying the nearest sequenced taxon for each feature sequence within this phylogenetic framework. Sequences with nearest-sequenced taxon index (NSTI) scores >2 were excluded. Gene family copy numbers per sample were subsequently calculated by integrating sequence abundance profiles. These gene families were mapped to the Kyoto Encyclopedia of Genes and Genomes (KEGG) database to quantify functional abundances. Complementarily, PICRUSt2 (17) was implemented for three-tiered metabolic pathway analysis, generating hierarchical abundance tables across three levels: broad functional categories, pathway subclasses, and specific biochemical reactions. Pathways demonstrating false discovery rate (FDR) <0.05 (Benjamini-Hochberg corrected) were considered statistically significant. LEfSe (18) was applied to quantitatively analyze biomarkers across different groups. The rfPermute R package (v2.5.4) (19) was implemented to construct a random forest classification model, utilizing differential abundant phylum and genus between healthy controls and RA patients as predictive features. Key parameters were set as ntree = 500 and nrep = 1000. For internal validation, a five-fold cross-validation protocol was repeated three times within the cohort. Classification performance was rigorously evaluated using the receiver operating characteristic (ROC) curve, with the area under the curve (AUC) providing a quantitative metric. The closer the AUC is to 1, the better the model performance.

### 2.3 Data sources for intestinal flora and RA

A two-sample MR study was employed to investigate the association between intestinal microbiota and RA occurrence. Multi-ethnic Genome-Wide Association Studies (GWAS) (20) data from 24 cohorts comprising 18,340 people formed the basis for our exposure factors. The dataset included 211 taxonomic units, comprising 131 species, 35 families, 20 orders, 16 classes, and 9 phyla. Additionally, the summary statistics about RA were derived from a GWAS dataset (ebi-a-GCST90013534), which included 43,923 controls and 14,361 cases (21). The initial GWAS accounted for population stratification by employing principal component analysis (PCA) and genomic control techniques. In this current study, we further refined our analysis by restricting the exposure (intestinal flora) and outcome (RA) data exclusively to individuals of European ancestry. The population selection, genotyping, and corresponding baseline data for the GWAS data involved in the study have been reported in previous research, and the original GWAS ethics committee approved the data collection.

### 2.4 Key Mendelian randomization assumptions

We systematically evaluated three core assumptions:

1. Relevance assumption: Instrumental variables (single nucleotide polymorphisms, SNPs) show strong association with exposure (*P* < 1 × 10^-5^) (22), with instrument strength confirmed by F-statistic >10.
2. Independence assumption: SNPs are independent of confounders, enforced through linkage disequilibrium (LD) clumping (r^2^ < 0.01, window size = 10 Mb) (23).
3. Exclusion-restriction assumption: SNPs influence RA solely through intestinal flora, with horizontal pleiotropy tested by non-significant MR-Egger intercept (*P* > 0.05).

### 2.5 Selection and exclusion of instrumental variables

Intestinal flora was classified at five taxonomic levels: genus, family, order, class, and phylum. Instrumental variables (IVs) were derived from SNPs meeting genome-wide suggestive significance (*P* < 1 × 10^-5^). To balance IV strength and independence, LD parameters were set at r^2^ < 0.01 with a 10 Mb clumping window (24). This stringent r^2^ threshold minimized LD bias while preserving statistical power. Finally, SNPs with ambiguous or palindromic alleles were excluded.

### 2.6 Mendelian randomization analysis

The causal relationship between intestinal flora and RA risk was evaluated using three MR methods (25). Inverse variance weighting (IVW) served as primary analysis, estimating causal effects through weighted genetic associations (26, 27). Weighted median (WM) provided robust estimation with invalid instruments, and MR-Egger regression detected directional pleiotropy via intercept testing (28, 29). Cochran’s Q test was used to quantify heterogeneity among IVs (*P* > 0.05 indicating no significant heterogeneity) (30–32). MR-PRESSO (33) was used to detect horizontal pleiotropy, and remove outliers. In addition, the leave-one-out analysis could examine whether a single SNP dominates the results. A symmetrical distribution of the funnel plot suggested the absence of directional pleiotropy. TwoSampleMR (v0.5.6) was used for quality control and instrumental variable selection. R software (v4.4.1) was utilized for data visualization and statistical analysis. The statistical significance threshold was set at *P* < 0.05 (24).

### 2.7 Animal housing and modeling

Male DBA/1J mice (8-week-old) were purchased from GemPharmatech Co., Ltd. in Jiangsu, China. All mice were kept in a particular pathogen-free environment with a 12:12 h light/dark cycle to establish a CIA model. The Animal Ethics Committee of Shanxi Medical University gave its approval to all of the research (2021–214).

DBA/1J mice were immunized on days 1 and 21 to construct the CIA model. In particular, the mice’s tails received an intradermal injection of 150 μg of type II bovine collagen (Chondrex, Redmond, WA, USA) emulsified in an equivalent volume of complete Freund’s adjuvant (Chondrex, Redmond, WA, USA). On day 21, 75 μg of CII emulsified in Freund’s incomplete adjuvant (Chondrex, Redmond, WA, USA) was administered as a second immunization. The following criteria were used to evaluate and score the mice’s toe and ankle joint inflammation every other day after the second vaccination: 0, normal; 1, mild finger erythema and swelling; 2, moderate ankle-to-tarsal erythema and swelling; 3, noticeable ankle-to-metatarsal joint swelling; 4, total ankle, foot, and finger swelling and redness, accompanied by deformity and/or ankylosis. The clinical evaluation was carried out in a blinded manner, and the sum of the scores for each limb was the overall clinical score (0–16).

### 2.8 Bacterial strain cultivation and administration

*Eubacterium rectale* JCM 17463 was purchased from Mingzhoubio (Zhejiang, China). The culture medium used was chopped meat carbohydrate broth. The bacteria were grown anaerobically for 48 h at 37°C in an anaerobic chamber (Bactron EZ-2, Shellab, USA). After that, the cells were collected for ten minutes at 5000 rpm. Anaerobic phosphate-buffered saline (PBS) was used to resuspend the bacterial suspension. A McFarland turbidity tube (1x10^9) was used to compare the bacterial suspension’s concentration. The culture was stored in 250 μL aliquots until used for gavage-feeding mice.

For bacterial administration, gavage feeding of the *E. rectale* group was initiated with *E. rectale* suspended in PBS every other day for 20 days after the appearance of joint symptoms; to the CIA group, 250 μl aliquots of PBS was given as gavage feeding every other day for 20 days after the appearance of joint symptoms.

### 2.9 Flow cytometry

After anesthetizing the mice, peripheral blood was collected by removing eyeballs and stored in EDTA-coated tubes. The mice were subsequently euthanized through cervical dislocation. To obtain a single-cell suspension, the spleen was removed by opening the abdominal cavity, then gently ground in PBS and filtered through a 40 μm cell strainer. Subsequently, mononuclear cells were isolated using an animal spleen lymphocyte isolation kit (Solebo, Beijing, China) to obtain a PBMC cell suspension. The cells were stained with anti-CD3 (Cat#563024, BD Pharmingen, USA), anti-CD4 (Cat#557307, BD Pharmingen, USA), anti-CD25 (Cat#566498, BD Pharmingen, USA), anti-Foxp3 (Cat#17-5773-82, Invitrogen, USA), anti-CXCR5 (Cat#145511, BioLegend, USA), and anti-PD-1 (Cat#561788, BD Pharmingen, USA). To eliminate debris, the cells were gated using forward scatter and side scatter (FSC/SSC). Data collection was performed using a BD flow cytometer (BD Biosciences, San Jose, CA, USA), and analyzed with FlowJo software.

### 2.10 Micro-computed tomography analysis

After euthanizing the mice, their ankle joints were harvested and fixed in 4% paraformaldehyde. Micro-CT analysis was carried out at 90 kV and 0.09 mA, achieving a resolution of 10 μm (Micro-CT, VNC-102, NEMO, China). The dataset was subsequently rebuilt to generate 3D pictures of the ankle joints. The ratio of bone surface to bone volume (BS/BV) and bone mineral density (BMD) were among the subsequent measurements of bone morphometric parameters.

### 2.11 Untargeted metabolomics analysis

Mouse serum and fecal samples stored at -80°C were thawed on ice. Subsequently, 50 μL of serum samples (or 20 mg of fecal samples) were mixed with 300 μL of extraction solution (acetonitrile: methanol = 1:4, v/v, with internal standards). After vortexing for 3 minutes, the mixture was centrifuged at 4°C/12,000 rpm for 10 minutes. Then, 200 μL of the supernatant was left to stand at -20°C for 30 minutes. After being thawed again, it was centrifuged at 4°C/12,000 rpm for 3 minutes. Finally, 180 μL of the supernatant was taken for instrumental analysis. Untargeted metabolomics employed the combination of chromatography and mass spectrometry for substance identification. Liquid chromatography-tandem mass spectrometry (LC-MS/MS) was performed using a Vanquish UHPLC system (Thermo Scientific, USA) coupled with a Q Exactive HF-X mass spectrometer (Thermo Scientific, USA). Raw data were converted to mzML format using ProteoWizard. Preprocessing (peak extraction, alignment, correction, and gap-filling) was conducted with XCMS, KNN, and SVR algorithms (34). Metabolite identification was achieved by querying Human Metabolome Database (HMDB) and KEGG databases, followed by KEGG pathway mapping for enrichment analysis. Differential metabolites were screened using Student’s t-test and orthogonal partial least squares-discriminant analysis (OPLS-DA). Metabolites with variable importance in projection (VIP) > 1 and *P* < 0.05 were considered statistically significant.

### 2.12 Statistical analysis

For all statistical studies, R software (version 4.4.1) was used. Group comparisons were performed using the t-test, and continuous data were reported using mean ± standard deviation. Pairwise comparisons were conducted using the LSD-t test, and group comparisons were conducted using the one-way analysis of variance test; if the variances were heterogeneous, the Mann-Whitney U test was employed. All P values were determined using two-sided testing, and *P* < 0.05 was taken as statistically significant.

## 3 Results

### 3.1 Changes in the diversity of intestinal microbiota in RA patients

**Figure 2:**
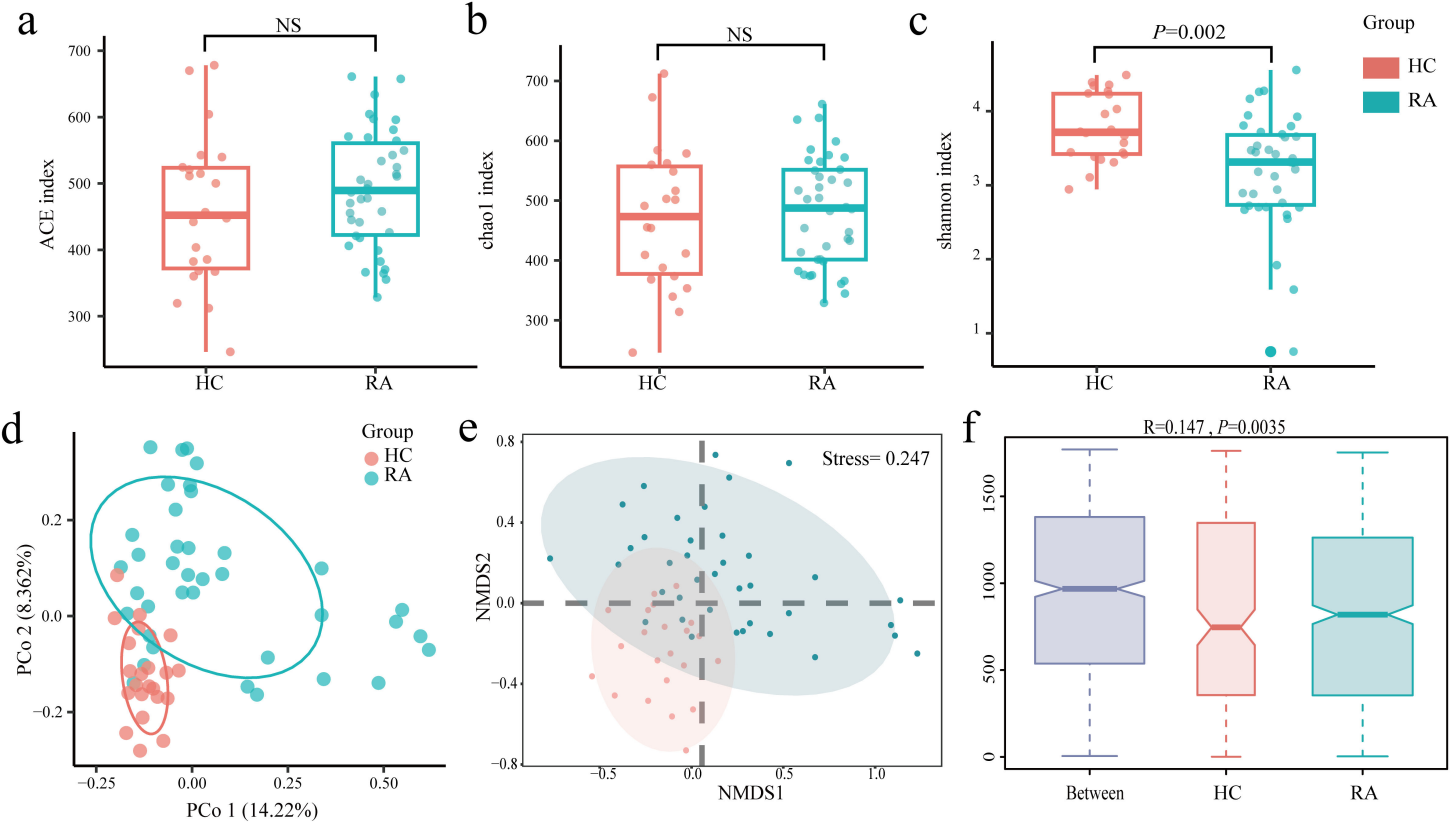
Changes in the gut microbiome between new-onset rheumatoid arthritis (RA) and healthy controls. **(a–c)** Alpha diversity between the HC group and the RA group was estimated by ACE, Chao1, and Shannon index. **(d–f)** Beta diversity between the HC group and the RA group was assessed by Bray-Curtis PCoA, NMDS, and ANOSIM. HC group, healthy control group; RA group, new-onset rheumatoid arthritis patient group.

The study used α and β diversity to assess how the intestinal flora diversity of the RA and HC groups differed. The Ace and Chao1 indices showed no statistically significant difference between the RA and HC groups (**Figures 2a, b**). The RA and HC groups did, however, differ statistically significantly, according to the Shannon index (*P* = 0.002) (**Figure 2c**). The differences between the HC and RA groups were further observed using PCoA with Bray-Curtis distance (**Figure 2d**). The composition of intestinal flora in new-onset RA patients and HC were found to differ significantly (R = 0.147, *P* = 0.0035), according to similarity analysis (ANOSIM), and Bray-Curtis’s NMDS revealed differences between the RA and HC groups (Stress = 0.247) (**Figures 2e, f**). These findings suggest significant diversity of intestinal flora in HC and new-onset RA patients.

### 3.2 Changes in the composition of the Intestinal flora in RA patients

**Figure 3:**
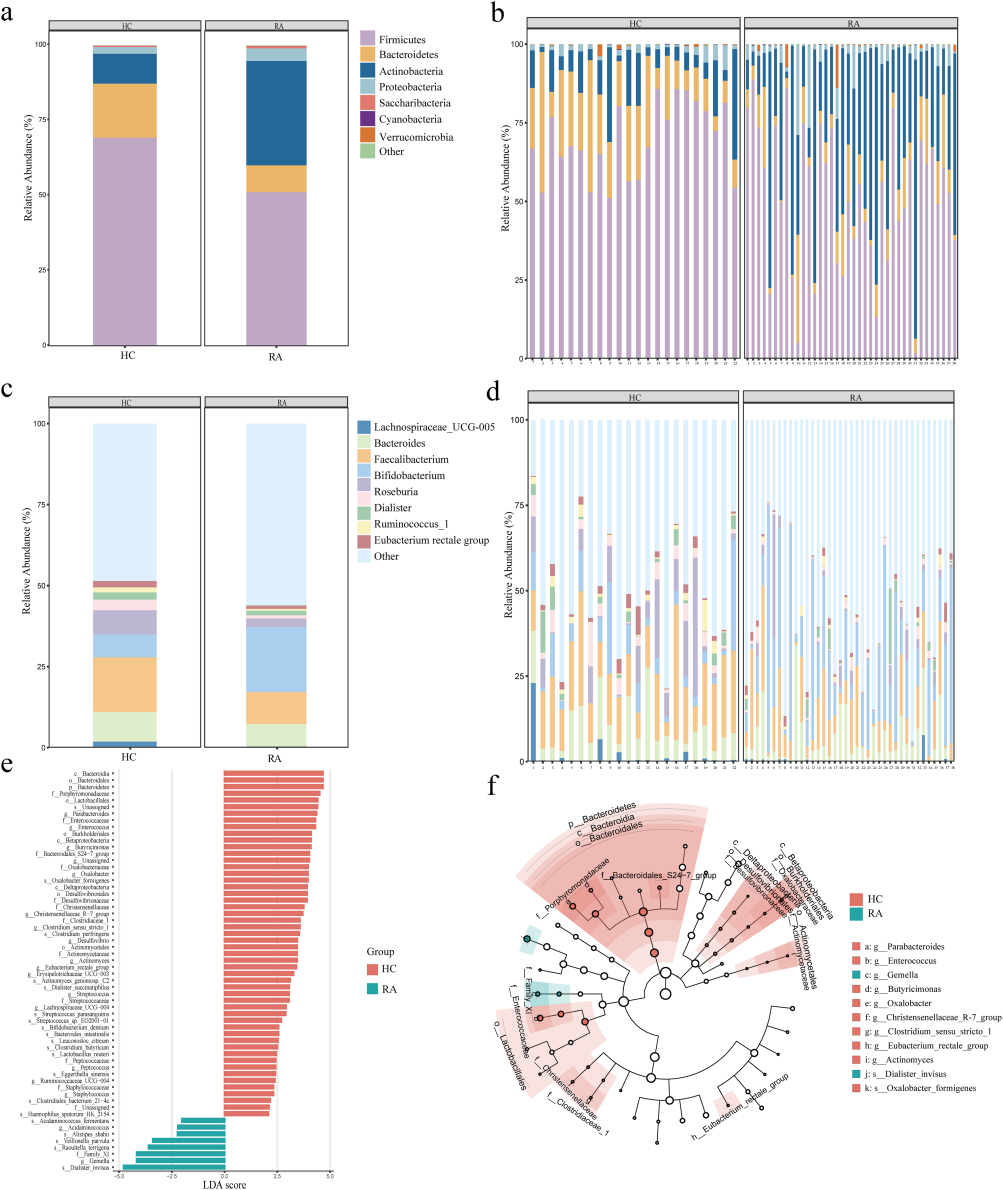
Alterations in the intestinal microbiota composition between newly diagnosed rheumatoid arthritis (RA) and healthy controls. **(a, b)** Phylum level (top 7) between HC and RA groups. **(c, d)** Genus level (top 9) between HC and RA groups. **(e)** Differential intestinal flora with LDA score > 3 between HC and RA groups, *P* < 0.05. **(f)** Phylogenetic distribution of the intestinal differential flora branch diagram between HC and RA groups. LDA: Linear Discriminant Analysis. HC group, healthy control group; RA group, new-onset rheumatoid arthritis patient group.

The proportions of dominating taxa between HCs and RA patients were examined at the genus and phylum levels. *Proteobacteria* and *Actinobacteria* were more common in RA patients than in the HC group at the phylum level, while *Firmicutes* and *Bacteroidetes* were less common (**Figures 3a, b**). *Bifidobacterium* was more prevalent in RA patients than in the HC group at the genus level. In contrast, RA patients had lower proportions of *E. rectale, Ruminococcus, Dialister, Roseburia, Pseudobutyrivibrio, Faecalibacterium, Bacteroides*, and *Lachnospiraceae* than the HC group (**Figures 3c, d**). To further examine the differences in intestinal flora between RA and HCs, linear discriminant analysis (LDA) was employed (LDA > 3, P < 0.05) effect size. At the genus level, we found that *Acidaminococcus* and *Gemella* were dominant in RA patients compared with HC (**Figures 3e, f**). At the phylum level, *Bacteroidetes* were relatively less dominant in RA than in HC (**Figures 3e, f**), and at the genus level, *Parabacteroides, Enterococcus, Butyricimonas, Oxalobacter, Christensenellaceae, Clostridium sensu stricto, Desulfovibrio, Actinomyces, Eubacterium rectale, Erysipelotrichaceae, Streptococcus, Lachnospiraceae, Ruminococcaceae, Peptococcus*, and *Staphylococcus* were less dominant in RA (**Figures 3e, f**).

### 3.3 Functional enrichment of Intestinal flora in RA patients

**Figure 4:**
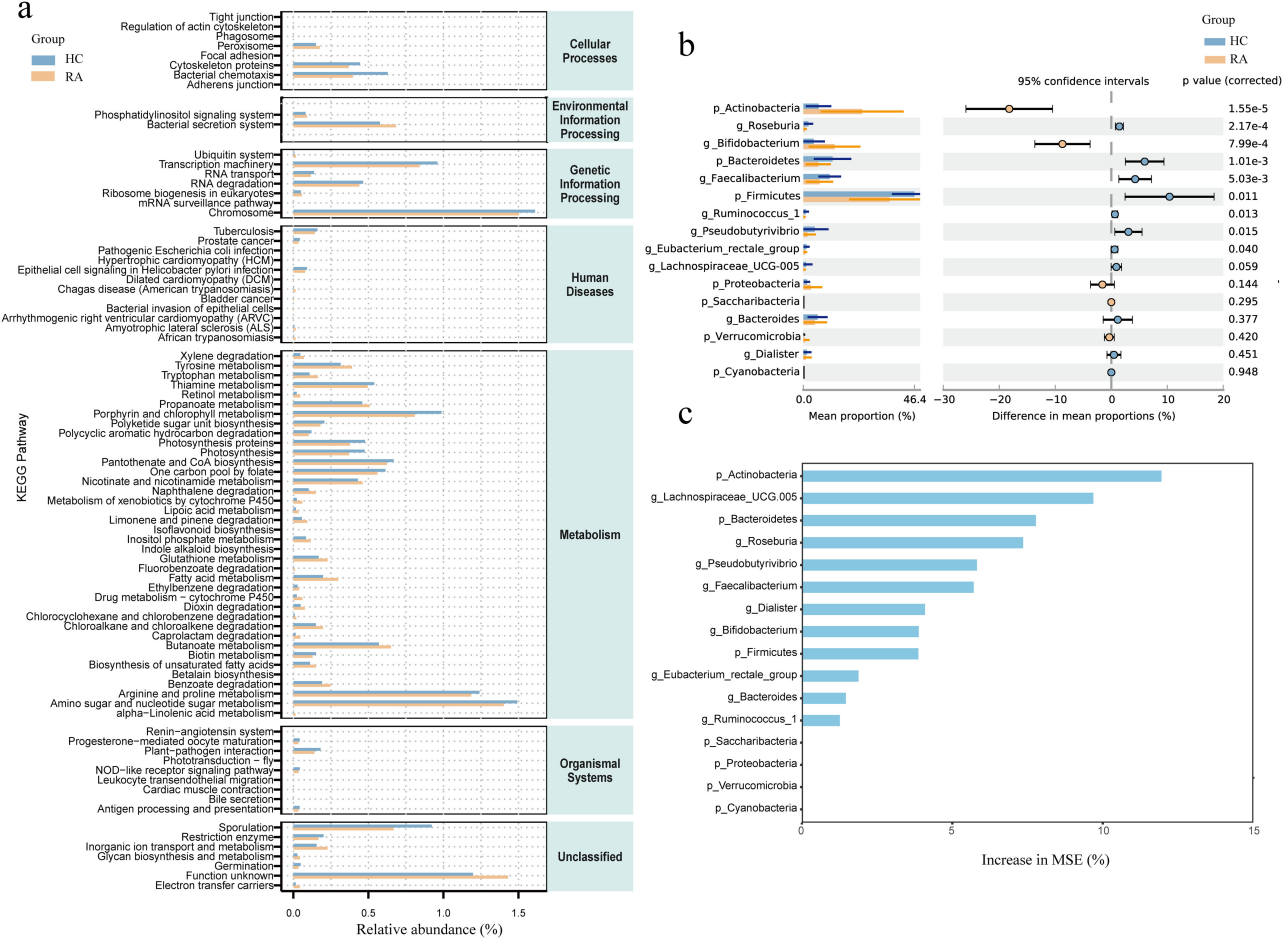
Comparison of differential intestinal microbiota between new-onset rheumatoid arthritis (RA) patients and healthy controls and functional enrichment of intestinal microbiota. **(a)** Functional enrichment analysis of microbiota between HC and RA groups. X-axis: Relative abundance (%); y-axis: Kyoto Encyclopedia of Genes and Genomes (KEGG) pathways. **(b)** Comparison of relative abundance of bacteria at phylum and genus levels between HC and RA groups. **(c)** Importance of random forest prediction features in HC and RA groups. Differences were considered statistically significant at *P* < 0.05. HC group, healthy control group; RA group, new-onset rheumatoid arthritis patient group.

The effects of intestinal flora on a number of biological processes, such as metabolism, organic systems, cellular processes, environmental information processing, human illnesses, genetic information processing, and unclassified categories, were predicted using PICRUSt2. We conducted a differential analysis using DESeq2 with an FDR threshold of < 0.01, identifying 83 functional pathways significantly differing between RA patients and HC groups. Some significant pathways were associated with metabolic processes, such as unsaturated fatty acid synthesis, fatty acid metabolism, butanoate metabolism, and biotin metabolism. There were also pathways associated with immune responses and signaling, including NOD-like receptor signaling, leukocyte transendothelial migration, and phosphatidylinositol signaling (**Figure 4a**).

**Figure 5:**
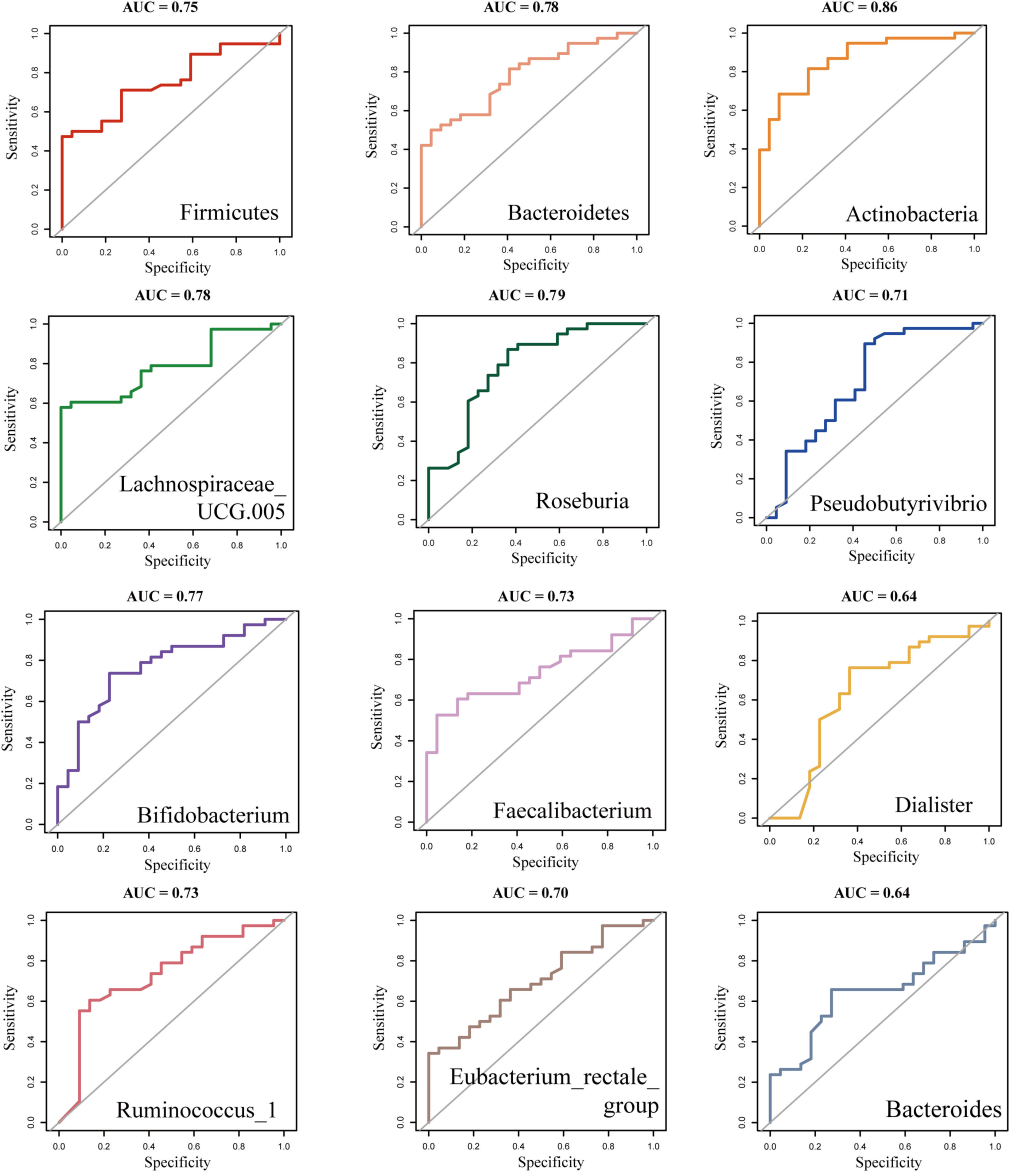
ROC analysis of differentially abundant Intestinal flora in new-onset rheumatoid arthritis (RA). ROC, Receiver operating curve.

We used the t-test to compare the differential bacterial communities between healthy subjects and RA subjects at the phylum and genus levels. *Actinobacteria* were more abundant in RA (*P* < 0.05) than in HC at the phylum level, while *Bacteroidetes* and *Firmicutes* were less abundant in RA (*P* < 0.05); at the genus level, *Bifidobacterium* was more abundant in RA (*P* < 0.05), while *Roseburia, Faecalibacterium, Ruminococcus, Pseudobutyrivibrio*, and *Eubacterium rectale* had lower relative abundances in RA (*P* < 0.05) (**Figure 4b**). We further constructed a random forest model using differential bacterial communities. The model showed that the more characteristic bacterial genera were *Firmicutes* (AUC = 0.75), *Bacteroidetes* (AUC = 0.78), *Actinobacteria* (AUC = 0.86), *Lachnospiraceae* (AUC = 0.78), *Roseburia* (AUC = 0.79), *Pseudobutyrivibrio* (AUC = 0.71), *Bifidobacterium* (AUC = 0.77), *Faecalibacterium* (AUC = 0.73), *Dialister* (AUC = 0.64), *Ruminococcus* (AUC = 0.73), *Eubacterium rectale* (AUC = 0.70), and *Bacteroides* (AUC = 0.64) (**Figures 4c**, **5**).

### 3.4 Causal relationship between intestinal microbiota and RA

**Figure 6:**
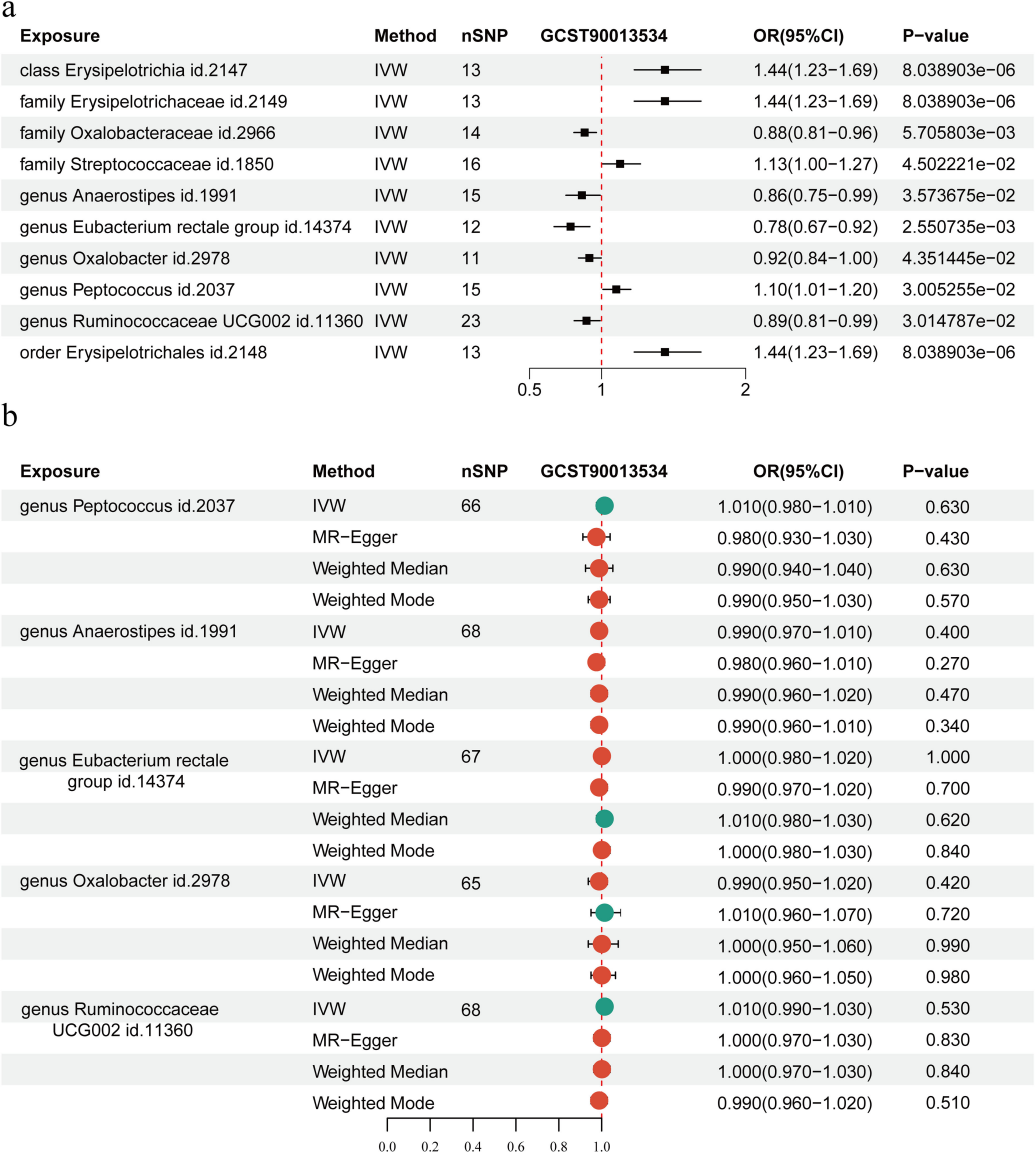
Mendelian randomization (MR) analysis of the causal relationship between Intestinal flora and rheumatoid arthritis (RA). **(a)** MR analysis of the causal relationship between Intestinal flora and RA. **(b)** MR analysis of the reverse causal relationship between intestinal microecology and RA. The Inverse Variance Weighting (IVW) method genetically predicts associations between RA and Intestinal flora. SNP, single nucleotide polymorphisms; OR, odds ratio; CI, confidence interval.

We examined the causal association between GWAS datasets linked to RA (ebi-a-GCST90013534) and 211 gut microbial taxa. Intestinal flora and RA were investigated using five different MR techniques: weighted median, MR Egger, simple mode, weighted mode, and IVW. The causal impact was measured using the odds ratio (OR), which shows that for every standard deviation rise in the abundance of intestinal flora features, there is a correspondingly higher risk of RA. IVs for each microbial taxonomic group comprised 3 to 23 SNPs, with mean F-statistics exceeding the critical threshold of 10 (range: 20.35-26.72), confirming the absence of weak instrument bias. The IVW analysis revealed that RA patients have a higher abundances of *Erysipelotrichia* (OR, 1.44; 95% CI, 1.23-1.69), *Erysipelotrichales* (OR, 1.44; 95% CI, 1.23-1.69), *Erysipelotrichaceae* (OR, 1.44; 95% CI, 1.23-1.69), *Peptococcus* (OR, 1.10; 95% CI, 1.01−1.20), and an elevated incidence of RA was substantially associated with *Streptococcaceae* (OR, 1.13; 95% CI, 1.00-1.27) at the class, order, family, and genus levels, respectively (*P* < 0.05) (**Figure 6a**). Conversely, higher abundances of *Oxalobacteraceae* (OR, 0.88; 95% CI, 0.81-0.96), *Anaerostipes* (OR, 0.86; 95% CI, 0.75-0.99), *Eubacterium rectale* (OR, 0.78; 95% CI, 0.67-0.92), *Oxalobacter* (OR, 0.92; 95% CI, 0.84-1.00), and *Ruminococcaceae UCG002* (OR, 0.89; 95% CI, 0.81-0.99) appeared to have a protective effect against RA (*P* < 0.05) (**Figure 6a**).

Finally, the association between intestinal microbiota and RA was examined using reverse MR based on IVW methods. According to our analysis, there was no reverse causal relationship between the risk of RA and *Ruminococcaceae* (P = 0.84), *Eubacterium rectale* (P = 1.00), *Oxalobacter* (P = 0.42), *Peptococcus* (P = 0.63), or *Anaerostipes* (P = 0.40) (**Figure 6b**).

### 3.5 Sensitivity and heterogeneity analysis between intestinal microbiota and RA

To evaluate the validity of our findings, we conducted sensitivity studies using the MR-Egger and WM methods. MR-PRESSO analysis suggested no horizontal pleiotropy between exposure and outcome for these taxa (**Supplementary Figure S1a**).

Cochran’s Q test showed minimal heterogeneity for the IVW method when evaluating the association between intestinal flora and the risk of RA, with low variability detected for *Peptococcus* (*P* = 0.47), *Anaerostipes* (*P* = 0.64), *E. rectale* (*P* = 0.11), *Oxalobacter* (*P* = 0.97), and *Ruminococcaceae* (*P* = 0.06) (**Supplementary Table S1**). After removing each SNP individually for testing, the results were like those obtained when all SNPs were included (**Supplementary Figure S1b**). Subsequently, the funnel plot did not reveal any asymmetry in the SNPs (**Supplementary Figure S1c**).

Cochran’s Q test showed that *Peptococcus* (*P* = 0.29), *Anaerostipes* (*P* = 1.00), *Eubacterium rectale group* (*P* = 0.89), *Oxalobacter* (*P* = 0.63), and *Ruminococcaceae* (*P* = 0.51) were significantly higher in the study of RA and intestinal. The relationships between microbial communities showed low heterogeneity (**Supplementary Table S2**).

### 3.6 The impact of *Eubacterium rectale* on arthritis in mice

**Figure 7:**
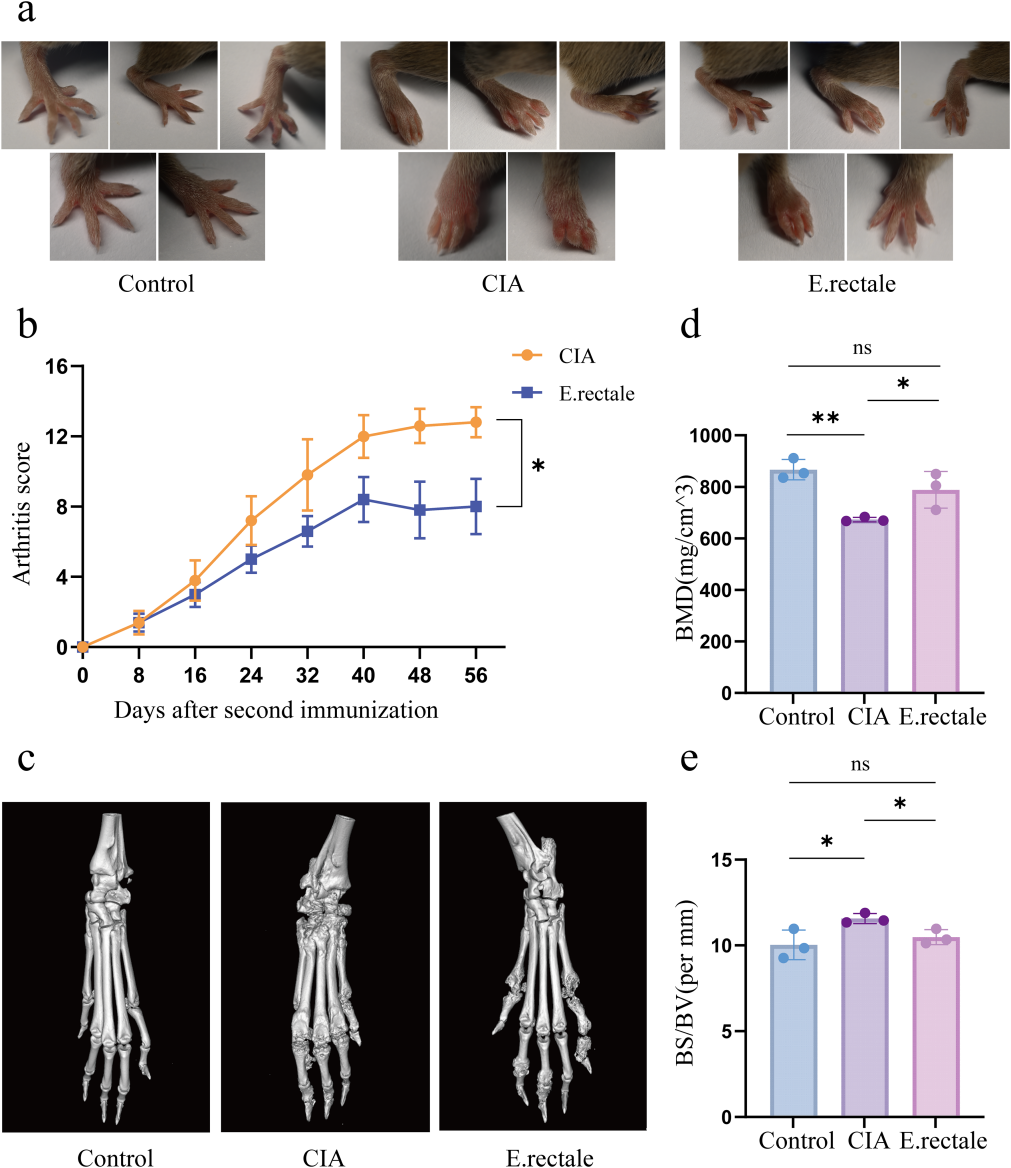
Joint manifestations in normal mice, CIA mice, and mice treated with *Eubacterium rectale* gavage. **(a)** Photographs of joint symptoms in each group of mice (n=5); **(b)** Levels of joint inflammation scores in each group of mice (n=5). **(c)** Representative micro-CT images of the ankle joints of mice in each group, showing the level of bone erosion in the ankle joints; **(d, e)** Quantitative micro-CT analysis of bone mineral density (BMD) and bone surface density (BS/BV) (n=3). Statistical analysis was performed using two-way ANOVA **(b)** and Student’s t-test (d, e). Data are presented as SD ± mean. *P < 0.05, **P < 0.01.

We used DBA/1 mice to develop a type II collagen-induced CIA mouse model to examine the potential role of *E. rectale* in RA. Two groups of CIA mice were treated with either *E. rectale* or PBS via gavage. The results showed that mice treated with *E. rectale* had lower arthritis scores and slower progression than the CIA group (**Figures 7a, b**). From each group, three mice were chosen at random for further bone quality studies of their ankle joints using micro-CT. The findings showed that CIA mice had significantly reduced BMD, rougher joint bone surfaces, and rougher bone erosion compared to healthy mice. In contrast, mice in the *E. rectale* group showed less bone erosion and higher BMD in their ankle joints, which were somewhat closer to those of healthy mice (**Figures 7c-e**). This suggests that gavage treatment with *E. rectale* reduced bone damage due to inflammation and delayed joint deterioration in mice.

### 3.7 The impact of *Eubacterium rectale* on the mouse immune system

**Figure 8:**
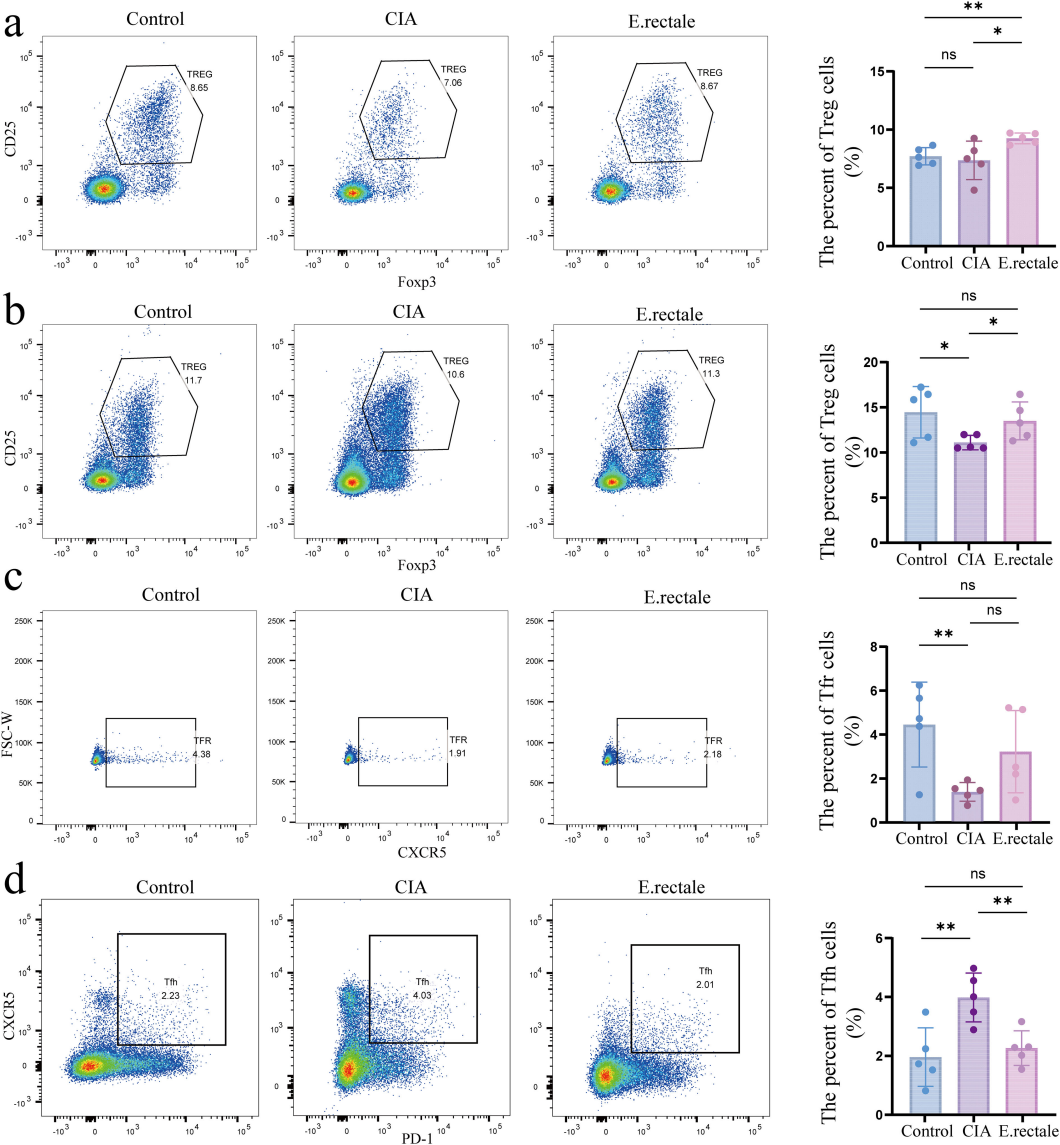
Flow cytometry was used to determine the proportions of Treg cells in peripheral blood and spleen and Tfr and Tfh cells in the spleen of different mice. **(a)** Representative flow cytometry results and statistical analysis of Treg cell proportions in peripheral blood (n=5); **(b)** Representative flow cytometry results and statistical analysis of Treg cell proportions in the spleen (n=5); **(c)** Representative flow cytometry results and statistical analysis of Tfr cell proportions in the spleen (n=5); **(d)** Representative flow cytometry results and statistical analysis of Tfh cell proportions in the spleen (n=5). Statistical analysis was performed using Student’s t-test, and data are presented as mean ± SD. Significance levels are indicated as *P < 0.05, **P < 0.01.

RA is an immune-related arthritic inflammatory disease. Considering the previously observed important role of immune cells, especially Treg and Tfr/Tfh cells, in mouse arthritis models, we performed flow cytometry to detect the proportion of immune cells in mice’s peripheral blood and spleen after different interventions. These cells included Tfh (CD3+CD4+CXCR5+PD-1+), Tfr (CD3+CD4+CXCR5+Foxp3+CD25+), and Treg cells (CD3+CD4+CD25+Foxp3+). The results showed a declining trend in Treg cells in the CIA mice’s spleen and peripheral blood as compared to the control group, which is consistent with previous research findings. However, compared to CIA mice, mice treated with *E. rectale* had considerably more Treg cells in their spleen and peripheral blood (**Figures 8a, b**). For Tfr cells in the spleen, CIA mice had significantly fewer cells than healthy mice (**Figure 8c**). In contrast, CIA mice had a significant increase in Tfh cells in the spleen, whereas animals treated with *E. rectale* exhibited a declining trend in comparison to CIA mice (**Figure 8d**). These findings suggest that gavage treatment with *E. rectale* improves collagen-induced immune imbalance and promotes the restoration of immune homeostasis, which may be the fundamental reason for improving arthritic lesions with *E. rectale* gavage treatment.

### 3.8 Metabolite alterations in CIA mice following *Eubacterium rectale* intervention

**Figure 9:**
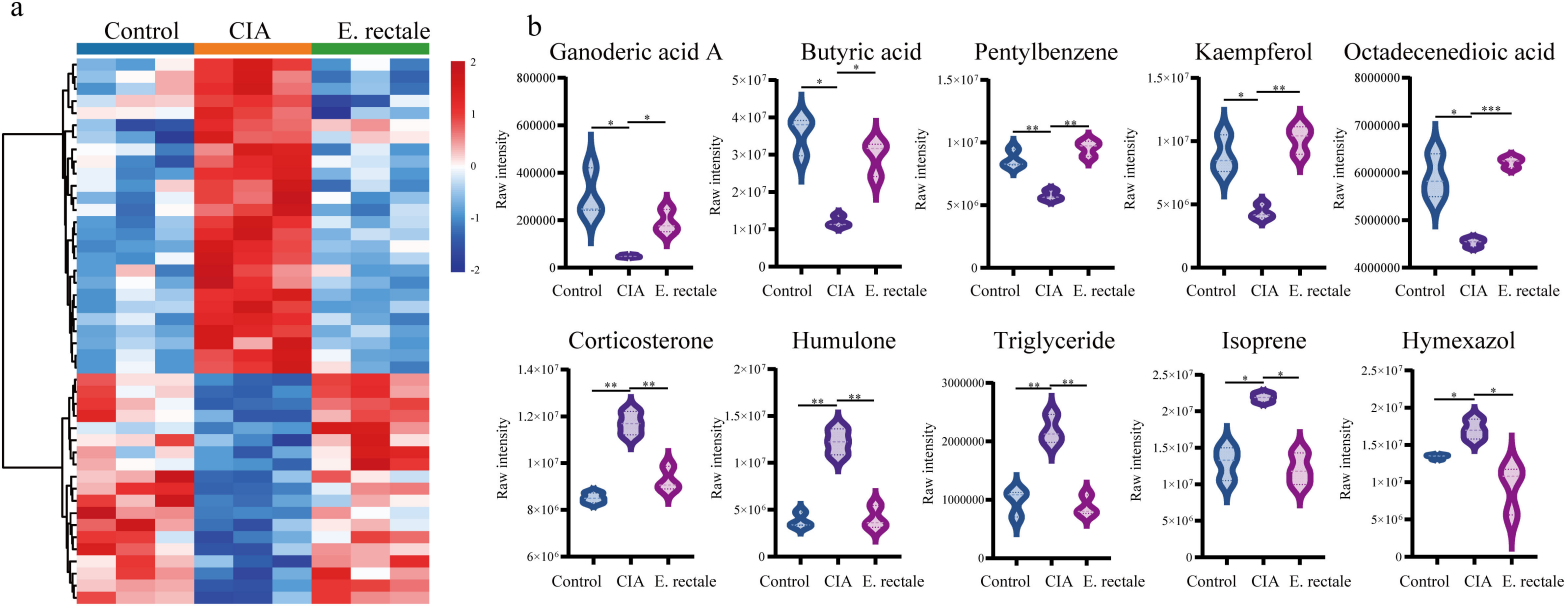
Untargeted metabolomics detection of changes in serum metabolites of different mice. **(a)** Changes in serum metabolites of healthy control group (Control group, n = 3), CIA mice (CIA group, n=3) and CIA mice treated with *Eubacterium rectale* (*E.rectale* group, n = 3); **(b)** Statistical analysis of the top ten differentially expressed metabolites among the three groups. Control group, n = 3; CIA group, n = 3; *E.rectale* group, n = 3. The significance levels are indicated by *P < 0.05, **P < 0.01 and ***P < 0.001.

Untargeted metabolomics analysis via LC-MS/MS was conducted on serum and fecal samples from three groups, healthy controls (n=3), CIA mice (n=3), and CIA mice treated with *E. rectale* (n=3). Serum metabolomics revealed that, versus healthy controls, the CIA group exhibited 95 upregulated and 105 downregulated metabolites. Treatment with *E. rectale* reversed these perturbations, showing 26 downregulated and 19 upregulated metabolites relative to the CIA group (**Figure 9a**; **Supplementary Table S3**). KEGG enrichment indicated that these metabolites were involved in tyrosine metabolism, vitamin digestion and absorption, fat digestion and absorption, gap junctions, protein digestion and absorption, ether lipid metabolism, phospholipase D signaling pathway (**Supplementary Figure S2a**). Top 10 differentially expressed metabolites included Corticosterone, Isoprene, Humulone, Hymexazol, TG, Butyric acid, Ganoderic acid A, 9-Octadecenedioic acid, Pentylbenzene, and Kaempferol 3-(2’’-acetylrhamnoside) (**Figure 9b**). In the fecal metabolomics study, compared with the healthy control group, 437 metabolites were upregulated and 162 metabolites were downregulated in the CIA group (**Supplementary Table S4**). Following *E. rectale* supplementation, there was a significant decrease in the levels of 239 metabolites concurrent with an increase in the levels of 37 metabolites compared to the CIA group (*P* < 0.05) (**Supplementary Figure S2b**). KEGG enrichment analysis demonstrated significant involvement of these metabolites in Steroid biosynthesis, Pantothenate and CoA biosynthesis, Inflammatory mediator regulation of TRP channels, Amino acid biosynthesis, Neutrophil extracellular trap formation, Vitamin B12 transport and metabolism, Tryptophan metabolism, Biosynthesis of valine, leucine, and isoleucine (**Supplementary Figure S2c**). Among them, Butyric acid showed a similar trend to that in the serum metabolome, being downregulated in the CIA group and showing an increasing trend after *E. rectale* treatment, with the difference being statistically significant.

## 4 Discussion

RA is an inflammatory, chronic systemic autoimmune disease that mostly affects the soft tissues that surround the joints. The two main characteristics are the presence of autoreactive T lymphocytes in the blood and synovial tissues and the generation of autoantibodies (35). Histologically, the hallmarks of RA are pannus formation, mainly synovial tissue hyperplasia, neovascularization, and infiltration of inflammatory cells (B cells, dendritic cells, fibroblasts, neutrophils, macrophages, activated T cells, plasma cells that produce autoantibodies, etc.). Left unchecked, it can cause irreversible damage to bones, cartilage, tendons, and ligaments. Intermittent joint pain and swelling are macroscopically indicative of this damage, which progressively worsens to joint deformity and disability (2, 4, 36). Many hypotheses exist regarding the pathogenesis of RA, and these are still under investigation. It is generally accepted that the onset of RA requires two factors: (1) genetic factors, including epigenetic modifications, genetic polymorphisms, etc. Certain environmental exposures, such as smoking, obesity, and certain pathogen infections, act as triggers for the production of autoreactive B cells and T cells due to genetic susceptibility; (2) these exposures provide activated antigen-presenting cells (APCs) to activate autoreactive lymphocytes that have already been generated, impairing immune tolerance and causing local harmful inflammatory responses (37). What is the intrinsic connection between these two factors or how they are triggered simultaneously remains a mysterious and fascinating question.

RA is a multi-stage process, and its related autoimmunity appears earlier than the onset of clinical diseases (38). Treg cells constitute a critical component of peripheral immune tolerance. A decrease in the number and/or activity of Treg cells in RA patients has been consistently shown by previous research findings and our preliminary studies. Inflammatory cytokines such as IL-6, TNF-α, and IL-17 are overproduced as a result of this loss, which leads to immunological imbalance and helps in the onset and progression of RA. But the majority of Tfh and Tfr cells, which control B-cell antigen detection, activation, and autoantibody synthesis, are found in the germinal center (GC). In the GC, Tfh cells specifically assist memory B-cell development, high-affinity antibody synthesis, and B-cell maturation. However, Tfr cells suppress the GC response by blocking the glucose metabolism of B cells and releasing anti-inflammatory mediators such TGF-β and IL-10, which have a persistently suppressive effect on the production of high-affinity autoantibodies. A lower Tfr/Tfh ratio results from RA’s increased Tfh cells and decreased Tfr cells. In individuals with RA, this ratio has an inverse relationship with serum anti-cyclic citrullinated peptide antibodies (ACPA), erythrocyte sedimentation rate (ESR), Disease Activity Score 28 (DAS28) index, and C-reactive protein (CRP).

Existing studies suggest that the initial site of autoantibody production may be far away from the joints, especially the mucosa of the periodontium, respiratory tract, and intestine. The human microbiome is an extensive and intricate ecosystem of microorganisms. The initial stage of local mucosal autoimmunity can gradually transition to systemic autoimmunity. Periodontal disease and changes in the spectrum of oral flora are prevalent for several years before the beginning of RA, and alterations in the RA patients’ gut microbiome have also been shown (39). One of the key environmental variables influencing the onset of RA is human microbes, which are a major source of antigens for the immune system. Because the colon contains many immune cells and a wide mucosal area, the intestinal microbiome is crucial in regulating the host’s immune response (40). The gut microbiome influences the immune system by changing the intestinal mucosa’s permeability, producing proinflammatory/anti-inflammatory metabolites, and bidirectional signaling with local immune cells. Abnormal intestinal flora structure increases the proportion of pathogens, increases proinflammatory metabolites, and damages the intestinal barrier, overactivating the innate immune system to reshape the immune landscape, which then drives aberrant adaptive immune system activation that results in RA (41). Potential therapeutic benefits for RA may result from interventions that target the intestinal microbiome of RA patients to change the microbiome’s variety and composition, manage it, and enhance the balance between immunological activity and intestinal homeostasis. Microbial therapy has great potential, and some positive attempts have been made. For instance, *L. paracasei* and *L. casei* are believed to diminish the severity of joint inflammation in RA model mice (7). Furthermore, in patients with active RA, multi-strain probiotic capsules containing *L. acidophilus* LA-14, *L. casei* LC-11, *L. lactis* LL-23, and *Bifidobacterium bifidum* BB-06, *Bifidobacterium lactis* BL-04, have demonstrated therapeutic potential (42).

Utilizing MR analysis and 16S rRNA gene sequencing on 38 RA patients and 22 healthy individuals, we looked at the differences in the intestinal microbiota between those with RA and those who were healthy. When comparing the intestinal microbiota of the RA group and the healthy control group at the phylum and genus levels, taxonomic analysis results showed significant differences; however, the alterations in the intestinal microbiota were mainly related to abundance rather than composition. *Proteobacteria* and *Actinobacteria* increased at the phylum level in RA patients, while the proportions of *Bacteroidetes* and *Firmicutes* had decreased. The differences in the intestinal flora of RA and HCs were more noticeable at the genus level: *Acidaminococcus*, which decomposes a variety of amino acids, mainly glutamate, and accumulates acetate and butyrate, and *Gemella*, a pathogenic intestinal bacterium, were relatively more abundant in RA patients. *Parabacteroides*, one of the main components of the intestinal flora of RA and healthy people, decreased in relative abundance, consistent with previous research results (43–45). We further constructed a random forest model using differential flora, which showed that nine bacteria, including *Firmicutes, Bacteroidetes, Actinobacteria, E. rectale*, and *Bacteroides*, were more characteristic (AUCs were all > 60%). Combined with the results of intestinal microbiome functional enrichment, the potential functions of differential microbiota are mainly concentrated in genetic information processing and material metabolism. They are mostly involved in chromosome regulation, RNA transcription and degradation, as well as the metabolism of fatty acids, amino acids, and sugars. This suggests that the potential mechanism of action of these different microbiota in exerting immune regulation may be related to epigenetic regulation and material metabolism signal transmission.

On this basis, to clarify whether there are bacteria in the differential microbiota that have a clear causal relationship with RA, we used the MR analysis method. The results showed that *Streptococcaceae* and *Erysipelotrichia* were linked to a higher incidence of RA. Conversely, *Anaerostipes*, *E. rectale*, *Oxalobacter*, and *Ruminococcaceae* are protective factors for RA. RA and the previously identified microbiota were not found to be causally related by the concurrent reverse Mendelian analysis. Interestingly, our 16S rRNA sequencing data revealed that *E. rectale*, which was found to have a reduced relative abundance in RA patients, was one of the differentially expressed bacteria with characteristic significance in the random forest model. According to earlier studies, *E. rectale* levels were also significantly lower in the feces of early RA patients (46). At the same time, the results of MR analysis suggested that it was a protective factor for RA, which deserves our attention. These results suggest that *E. rectale* is one of the effective indicators to evaluate the intestinal microecological health level of patients with RA, and has great potential in developing intestinal regulatory treatment strategies for RA.

*E. rectale* is an important anaerobic Gram-positive bacterium belonging to the phylum *Firmicutes* and the family *Lachnospiraceae*. Because of its ability to produce butyrate, a short-chain fatty acid essential for preserving intestinal health and regulating systemic immunological activity, this bacterium has attracted a notable attention. *E. rectale* has been linked in several studies to autoimmune conditions, including psoriasis, inflammatory bowel disease, multiple sclerosis, and psoriatic arthritis (47–50). *E. rectale* was used in our study to treat mice with CIA. The results indicated that the arthritis score levels of the mice after intervention were significantly lower, and the local bone destruction in the joints was also less severe. This confirms that *E. rectale* improves joint inflammation in CIA mice to a certain extent. Considering the important role of *E. rectale* found in various immune-related diseases, we further evaluated the immune system of these mice using flow cytometry and found that the proportion of Tregs increased and the imbalance of Tfr/Tfh cells recovered in mice treated with *E. rectale*. This gives us reason to believe that *E. rectale* has an important role in regulating the immune function of RA patients. This phenomenon may be closely related to the butyric acid production ability of *E. rectale* butyrate can maintain immune tolerance and promote the differentiation of regulatory T cells (51, 52). When the number of *E. rectale* decreases, it leads to increase in proinflammatory cytokines (such as IL-6 and TNF-α). In addition, the lack of butyrate weakens the intestinal barrier’s integrity, which may make it easy for the antigenic substances in the intestine to trigger systemic autoimmune reactions (53, 54). However, our study has inherent limitations. First, absence of inactivated bacterial controls precludes definitive exclusion of non-viable component effects. In addition, although increased butyrate content was observed in the *E. rectale* treated group, we cannot exclude the contribution of other metabolites given the multienzyme synthesis capacity of *E. rectale*. Finally, functional differences between the mouse microbiome and the human microbiome may limit direct clinical extrapolation. These preclinical findings require further validation in human studies.

## 5 Conclusion

We examined 16S rRNA sequencing data and GWAS databases to further validate the causal relationship between alterations in the intestinal flora and RA. This analysis identified the *E. rectale* as one of the key intestinal flora associated with RA. *In vivo* experiments in a mouse model demonstrated that intragastric administration of *E. rectale* ameliorated CIA arthritis and reversed associated immune imbalances, suggesting it may represent a potential therapeutic candidate for rheumatoid arthritis.

## Data Availability

The original contributions presented in the study are publicly available. This data can be found here: http://www.ncbi.nlm.nih.gov/bioproject/1294269.
