# Supplementary material for: From gut to joint: the protective impact of Eubacterium rectale on rheumatoid arthritis
Source: Front Immunol. 2025 Jul 25;16:1607804. doi: 10.3389/fimmu.2025.1607804 (PMC12331731; doi:10.3389/fimmu.2025.1607804)
Supplement: Supplementary file 1 [file DataSheet1.pdf]

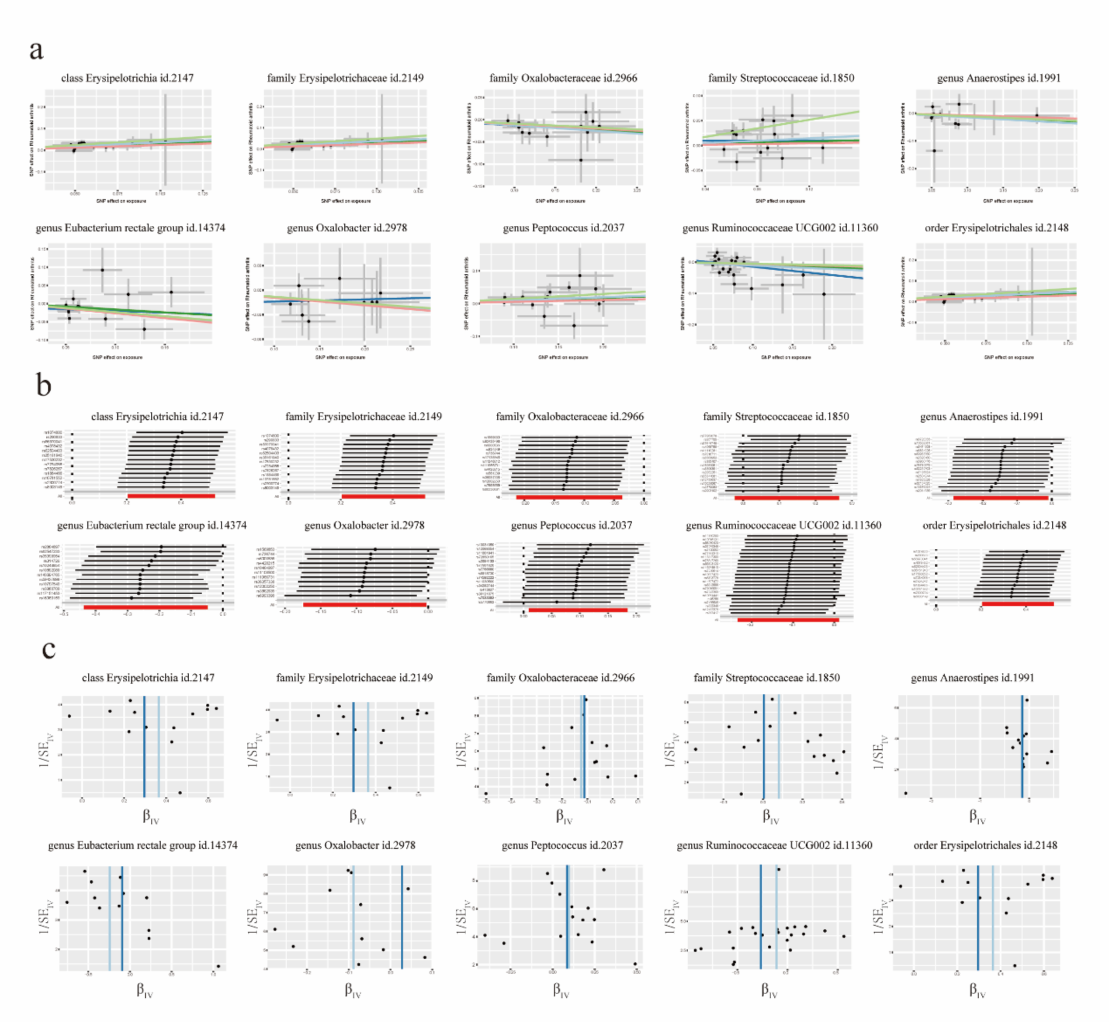

Figure S1: Mendelian randomization (MR) analysis of the causal relationship between Intestinal flora and rheumatoid arthritis (RA). (a) Scatter plots illustrating the relationship between RA risk and Intestinal flora. (b) Stepwise exclusion is used in a forest plot to show how individual single nucleotide polymorphisms (SNPs) affect RA. The leave-one-out analysis showed no marked difference in causal estimations of each Intestinal flora on RA. (c) Funnel plot assessing heterogeneity, with the blue line representing IVW and the dark blue line representing MR-Egger. SNP: single nucleotide polymorphisms; OR: odds ratio; CI: confidence interval.

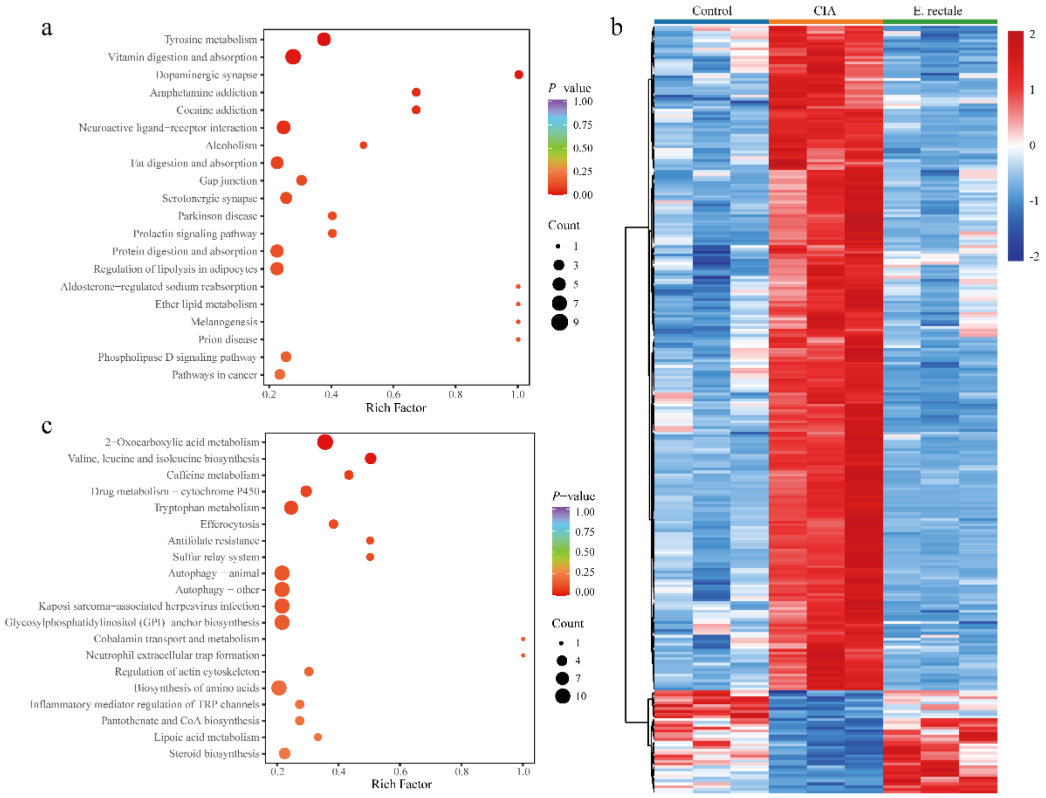

Figure S2: Metabolomics was used to detect the changes in serum and fecal metabolites and the enrichment of differential metabolite signaling pathways in different mice. (a) Differential serum metabolite-related signaling pathway enrichment among healthy control group (Control group, n = 3), CIA mice (CIA group, n=3) and CIA mice treated with *Eubacterium rectale* (*E.rectale* group, n = 3). (b) Differential fecal metabolites among control group (n = 3), CIA group (n = 3), *E.rectale* group (n = 3). (c) Differential fecal metabolite signaling pathway enrichment among control group (n = 3), CIA group (n = 3), *E.rectale* group (n = 3).

Table S1: Cochran's Q Test for Heterogeneity in Inverse Variance Weighting (IVW) and MR-Egger Methods in Exploring the Association Between Intestinal flora and Rheumatoid Arthritis (RA).

Exposure	Outcome	N.SNPs	F-statistic	IVW				MR-Egger			
				OR(95%CI)	*P*	Qstatistic	Q_pval	OR(95%CI)	*P*	Intercept	P_{inter}
Erysipelotrichia	RA	13	22.10	1.44(1.23-1.69)	<0.001	6.88	0.87	1.35(0.57-3.17)	0.51	0.004	0.88
Erysipelotrichaceae		13	22.10	1.44(1.23-1.69)	<0.001	6.88	0.87	1.35(0.57-3.17)	0.51	0.004	0.88
Oxalobacteraceae		14	22.63	0.88(0.81-0.96)	0.01	5.38	0.97	0.89(0.61-1.31)	0.58	-0.002	0.95
Streptococcaceae		16	22.82	1.13(1.00-1.27)	0.05	22.31	0.10	1.01(0.51-1.99)	0.98	0.009	0.74
Anaerostipes		15	21.79	0.86(0.75-0.99)	0.04	11.62	0.64	0.87(0.60-1.25)	0.47	-0.001	0.97
Eubacterium rectale group		12	21.61	0.78(0.67-0.92)	<0.001	16.77	0.11	0.91(0.49-1.69)	0.77	-0.010	0.63
Oxalobacter		11	23.84	0.92(0.84-1.00)	0.04	4.07	0.94	1.03(0.65-1.62)	0.91	-0.017	0.62
Peptococcus		15	22.90	1.10(1.01-1.20)	0.03	13.67	0.47	1.09(0.77-1.54)	0.63	0.001	0.95
Ruminococcaceae		23	22.07	0.89(0.81-0.99)	0.03	32.97	0.06	0.77(0.53-1.10)	0.17	0.012	0.39
Erysipelotrichales		13	22.10	1.44(1.23-1.69)	<0.001	6.88	0.87	1.35(0.57-3.17)	0.51	0.004	0.88

Table S2: Cochran's Q Test for Heterogeneity in Inverse Variance Weighting (IVW) and MR-Egger Analysis of the Reverse Association Between Intestinal flora and Rheumatoid Arthritis (RA).

Exposure	Outcome	N.SNPs	F-statistic	IVW				MR-Egger			
				OR(95%CI)	*P*	Qstatistic	Q_pval	OR(95%CI)	*P*	Intercept	P_{inter}
RA	Peptococcus	66	123.37	1.01(0.98-1.04)	0.63	70.70	0.29	0.98(0.93-1.03)	0.43	0.006	0.17
	Anaerostipes	68	130.51	0.99(0.97-1.01)	0.40	34.68	1.00	0.98(0.96-1.01)	0.27	0.002	0.47
	Eubacterium rectale group	67	131.67	1.00(0.98-1.02)	1.00	52.06	0.89	0.99(0.97-1.02)	0.70	0.001	0.61
	Oxalobacter	65	124.36	0.99(0.95-1.02)	0.42	59.66	0.63	1.01(0.96-1.07)	0.72	-0.005	0.26
	Ruminococcaceae	68	130.51	1.00(0.98-1.02)	0.84	66.06	0.51	1.03(1.00-1.06)	0.05	-0.006	0.02

Table S3: Untargeted metabolomics to detect changes in serum metabolites in different mice.

Index	Compounds	CIA_vs_HC_VIP	CIA_vs_HC_P-value	CIA_vs_HC_Type	E_vs_CIA_VIP	E_vs_CIA_P-value	E_vs_CIA_Type
MW0009449	Phthalic acid	1.653753942	0.023929234	up	1.504354	0.049667	down
MEDP1639	Corticosterone	1.791729527	0.003155672	up	1.700263	0.004249	down
MW0169682	Piperidine	1.680970304	0.01487842	up	1.508761	0.032317	down
MW0109260	Phosphonoacetate	1.615118328	0.006019441	up	1.779171	0.000385	down
MW0016270	Butyric acid	1.777420815	0.010942116	down	1.743838	0.016405	up
MEDP1577	Norvaline	1.525672844	0.036458037	up	1.683126	0.005896	down
MW0110915	Arachidonoyl Thio-PC	1.671900607	0.005960814	down	1.727647	0.020088	up
MW0118291	2-Aminopyrazine	1.663990814	0.009186107	down	1.592629	0.027417	up
MW0014686	5alpha-Androstane-3alpha,17beta-diol	1.562225301	0.028560404	up	1.650602	0.011693	down

MW0009692	Rosiglitazone	1.388630063	0.020594642	up	1.485074	0.02431	down
MW0109730	S-Methyl 2-propene-1-sulfinothioate	1.600312793	0.020992937	up	1.520025	0.038348	down
MW0005467	Hesperetin	1.531462754	0.016153737	down	1.40832	0.046417	up
MW0116159	AM2201 N-(3-fluoropentyl) isomer	1.554623286	0.030878185	down	1.554109	0.014488	up
MW0151857	ISOPRENE	1.697929395	0.016135892	up	1.706311	0.011699	down
MEDP1318	LPC(0:0/22:4)	1.665085616	0.008655454	up	1.736263	0.007596	down
MW0150217	Glu-Val-Phe-Glu	1.663211747	0.012284863	up	1.697636	0.005091	down
MW0154136	N1,N5,N10-tricaffeoyl spermidine	1.670800159	0.002112149	up	1.694446	0.003892	down

MW0122340	7-[3-(Dimethylamino)propoxy]-6-Methoxy-2-(4-Methyl-1,4-Diazepan-1-Yl)-N-(1-Methylpiperidin-4-Yl)quinazolin-4-Amine	1.603443426	0.022724314	down	1.591708	0.045285	up
MW0146115	Asp-Gly-Lys-Ala-Asp	1.686909979	0.040653198	up	1.656777	0.035134	down
MW0053975	Hydrocortisone acetate	1.48164152	0.04989426	up	1.528273	0.035242	down
MW0015586	Adrenic acid	1.655016923	0.012022632	up	1.622257	0.01624	down
MW0114601	Humulone	1.7806384	0.002143098	up	1.708409	0.00166	down

MEDL02162	FFA(22:5)	1.489432245	0.049658249	up	1.688803	0.005919	down
MW0149467	Ganoderic acid A	1.786601978	0.048726835	down	1.75188	0.035841	up
MW0124365	Hymexazol	1.69115014	0.036977474	up	1.439096	0.041428	down
MW0103061	FAA(18:1)	1.515535655	0.043601902	down	1.643616	0.031288	up
MW0062149	Prednisolone	1.622331435	0.029493324	up	1.630404	0.025791	down
MEDN0648	2,5-Dihydroxybenzaldehyde	1.451659498	0.033546387	down	1.478904	0.041249	up
MW0154184	N-Acetoxy-meiqx	1.666231858	0.005083995	up	1.646234	0.00601	down
MW0104373	2-[1-(Carboxymethyl)-3-methylcyclohexyl]acetic acid	1.578566665	0.044374046	down	1.484066	0.041126	up

MW0156094	Propan-2-yl 7-{(1R,2R,3S,5S)-2-[(3R)-3-(2,3-dihydro-1H-inden-2-yl)-3-hydroxyprop-1-en-1-yl]-3-fluoro-5-hydroxycyclopentyl}hept-5-enoate	1.685769979	0.011094432	down	1.701696	0.002658	up
MW0147336	Cerberin	1.601246986	0.02267349	down	1.597405	0.019231	up
MW0154695	9-Octadecenedioic acid, (9Z)-	1.725215699	0.031542989	down	1.792376	4.39E-05	up

MW0009234	N-cyclopropyl-8-[3-hydroxy-5-(2-methyloctan-2-yl)phenoxy]octanamide	1.249554964	0.043920498	up	1.577562	0.013707	down
MW0168304	Docosa-4,10,13,16-tetraenoic acid	1.50155121	0.043131657	up	1.703012	0.005322	down
ZINC1699444	Pentylbenzene	1.757076038	0.0078497	down	1.773226	0.002045	up
MW0054494	LPA(20:2(11Z,14Z)/0:0)	1.672976758	0.008506681	up	1.552983	0.023644	down
MW0124099	Febuxostat	1.697570917	0.030494657	down	1.592604	0.02999	up
MW0056309	PA(22:5(4Z,7Z,10Z,13Z,16Z)/20:0)	1.646567258	0.004858352	down	1.657024	0.049878	up

MW0056454	1,2-Dioctanoyl-sn-glycero-3-phosphate	1.679706804	0.003747669	up	1.721606	0.009683	down
MW0012841	1-Hydroxypropan-2-yl 2-isopropyl-5-methylcyclohexyl carbonate	1.588477854	0.022931475	down	1.648348	0.014697	up
MW0012955	1-Palmitoyl-2-arachidonoyl-sn-glycero-3-phosphoglycerol	1.560974853	0.032242412	down	1.599793	0.005401	up

MW0138595	Kaempferol 3-(2"-acetylrhamnoside)	1.734471123	0.023323151	down	1.761086	0.004062	up
MEDN1266	LPE(22:5/0:0)	1.489846022	0.039615283	up	1.645776	0.00942	down
MW0064059	TG(10:0/18:0/8:0)	1.70682752	0.003385009	up	1.743982	0.002486	down

Table S4: Untargeted metabolomics to detect changes in fecal metabolites in different mice.

Index	Compounds	CIA_vs_HC_VIP	CIA_vs_HC_P-value	CIA_vs_HC_Type	E_vs_CIA_VIP	E_vs_CIA_P-value	E_vs_CIA_Type
MEDP0603	Cuminaldehyde	1.627438419	0.025390609	up	1.415191956	0.023879678	down
MEDN0479	4-Methylvaleric Acid	1.312451031	0.041917846	up	1.357807371	0.036059203	down
MW0010797	5,6-EET	1.565171383	0.047838025	down	1.429609271	0.005708044	up
MEDP1061	o-Tyrosine	1.719279719	0.002974864	up	1.270817522	0.04356656	down
MW0052535	Enoxolone	1.712225512	0.0009237	up	1.386001728	0.001432853	down
MW0102800	thromboxane B2	1.383047998	0.03562431	up	1.231429672	0.034093631	down

MW0062278	Prostaglandin K1	1.515990775	0.023593456	up	1.413155824	0.032222966	down
MW0000273	Harmaline	1.484063212	0.015152167	up	1.376365085	0.015841052	down
MEDL01948	Jasmone	1.640357964	0.039003574	up	1.249712345	0.030265356	down
MEDP0577	Carnitine isoC4:0	1.102971479	0.048925997	up	1.262212844	0.028932231	down
MEDP2209	Meestrol	1.553310227	0.013828038	down	1.375708834	0.017570722	up
MW0146999	Calcium pantothenate	1.080348858	0.04337657	up	1.182661416	0.045557599	down
MW0006346	Betaxolol	1.426301834	0.033390963	up	1.435903667	0.025777178	down
MEDP1139	Linoleylethanolamide	1.574164806	0.015612759	up	1.257074172	0.043988124	down
MW0007503	Lidocaine	1.442446634	0.002324266	up	1.464110955	0.001669359	down
MW0009348	(+)-Penbutolol	1.620305602	0.006956262	up	1.469073854	0.01164896	down
MW0055241	N-acetylsphingosine	1.512142526	0.031728847	up	1.262537787	0.037606951	down
MEDP1194	Calcitriol	1.439330896	0.041135995	up	1.454736959	0.00149044	down
MW0000280	Heliotrine	1.440875138	0.016169172	up	1.479548779	0.000281692	down

ZINC6845904	Squalene	1.294928193	0.044064582	up	1.410696821	0.017864249	down
MW0145241	(4S,4aS)-1-amino-7-ethenyl-4,7-dimethyl-4,4a,5,6-tetrahydropyrrolo[1,2-c]pyrimidin-3-one	1.250269038	0.043602642	up	1.257602412	0.025608881	down
MW0000336	Methysergide	1.593144092	0.019761664	up	1.296901972	0.020630158	down
MEDP1665	Carnitine C3:0	1.460204067	0.009928109	up	1.419491752	0.004956494	down
MW0006033	Amitriptyline	1.510281412	0.043679864	up	1.317096785	0.031324055	down
MW0124782	Meperidine	1.564838698	0.026646428	up	1.383074904	0.033786435	down
MW0148895	Endomorphin-1	1.723037743	0.035014945	down	1.472950561	0.036801355	up
MW0007578	Melamine	1.499691073	0.044285011	up	1.480275976	0.00126695	down
MW0169483	Milrinone	1.420637037	0.033373398	up	1.32732724	0.03441396	down
MW0000258	Ethylmorphine	1.459039304	0.026906984	up	1.217969236	0.038286955	down
MW0138816	Lunarine	1.634819345	0.03224168	down	1.433702356	0.029829433	up
MW0007455	Karbutilate	1.618187831	0.003193782	up	1.376256453	0.003088169	down
MW0124515	Isouron	1.701036958	0.043661505	up	1.394614373	0.043174376	down

MW0015242	7alpha,12alpha-Dihydroxy-5beta-cholestan-3-one	1.729569557	0.026498252	up	1.399143139	0.030733246	down
MW0012107	11-deoxy-PGE2	1.450100588	0.011288382	up	1.419235466	0.02284568	down
MW0112095	Crotyl alcohol	1.681600279	0.017182859	up	1.43327556	0.017372601	down
MW0141191	1,8-Diazacyclotetradecane-2,9-dione	1.408472657	0.041472332	up	1.301089696	0.046308255	down
MW0126281	Purmorphamine	1.661627389	0.001930181	up	1.45562322	0.003942903	down
MW0011870	1,2-Dipalmitoyl-rac-glycerol	1.397650916	0.047960856	down	1.255087329	0.005341296	up
MW0062174	previtamin D3	1.612682228	0.012139055	up	1.243401818	0.040349202	down
MW0151243	Hydrocortisone cypionate	1.552786271	0.015830469	up	1.420721332	0.018390114	down
MW0052592	Equilin	1.546793908	0.022253614	up	1.118947937	0.043493718	down
MW0148928	ent-Corey PG-Lactone Diol	1.646177612	0.022311362	up	1.432803014	0.013289657	down
MW0169049	Deoxypeganine	1.278005485	0.025722248	up	1.340223134	0.024711396	down
MW0013712	beta-Boswellic acid acetate	1.224236021	0.031686017	up	1.457996536	0.019738221	down
MW0124681	Linalyl oxide	1.690018173	0.049498581	up	1.268222884	0.046998538	down

MW0049211	delta-Tocotrienol	1.71728932	0.0172478	up	1.465847956	0.02061038	down
FDATN01120	Marimastat	1.452108635	0.013079896	up	1.452643041	0.014413694	down
MW0009749	Sinequan	1.494139693	0.013609717	up	1.479064986	0.022724639	down
MW0108870	N-Octadecyl-N'-propyl-sulfamide	1.723806281	0.016885869	up	1.467908042	0.016347846	down
MW0145149	Ansamitocin P-3	1.432945289	0.020852426	up	1.434154386	0.021337052	down
MW0012743	19-Noretiocholanolone	1.532410594	0.043611528	down	1.378193309	0.027160596	up
MW0000329	Maraviroc	1.618880118	0.015801177	down	1.347374192	0.044267029	up
MW0055293	Nervonic acid	1.707378732	0.031483135	up	1.362746137	0.01492905	down
MW0006091	Atipamezole	1.338689765	0.022072749	up	1.469278147	0.008993921	down
MW0009652	Ranolazine	1.511714656	0.020786092	up	1.181706885	0.027679575	down
MW0122760	Alcaftadine	1.561698361	0.003517899	up	1.222593325	0.018009136	down
MW0063596	Sorbitan, monohexadecanoate	1.712443934	0.00989606	up	1.33242969	0.009604392	down
MW0063576	Sarsasapogenone	1.62369223	0.001114759	up	1.468567271	0.002323733	down

MW0159964	2-(ethylamino)-6-(propan-2-ylamino)-1H-1,3,5-triazin-4-one	1.539858848	0.027090881	up	1.33290818	0.040625366	down
MW0129716	1,7-Diphenyl-4-hepten-3-one	1.571901457	0.006179494	up	1.466855016	0.019545972	down
MW0109152	Phe-arg	1.634622839	0.043775556	up	1.225259273	0.045354728	down
MW0061079	5,6-Epoxy-5,6-dihydro-12'-apo-b-carotene-3,12'-diol	1.523582993	0.017143791	up	1.260614432	0.017059881	down
MW0169591	(2-Hydroxy-2-oxo-1,2lambda5-oxaphospholan-5-yl)methyl (Z)-octadec-9-enoate	1.554639814	0.015873731	up	1.422714878	0.019249239	down
MW0001653	1-Naphthylamine	1.593750693	0.0125597	up	1.362592886	0.045193297	down
MEDP1662	N6,N6,N6-Trimethyl-L-lysine	1.631801942	0.004261722	up	1.416003381	0.006857463	down
MW0120702	7-(2-(2-(Dimethylamino)ethoxy)ethoxy)-6-methoxy-2-(4-methyl-1,4-diazepan-1-yl)-N-(1-methylpiperidin-4-yl)quinazolin-4-amine	1.716960656	0.02162099	up	1.476739473	0.02018857	down
MW0151556	Ile-Pro-Asp	1.683286303	0.044735453	up	1.395798826	0.04817222	down
MW0155984	Pro-Pro-Thr	1.502410497	0.006024492	up	1.41527195	0.005465654	down
MW0123736	Disopyramide	1.610754914	0.03357604	up	1.352380555	0.049707764	down
MW0156323	R-4-benzyl-3-((R)-3-hydroxy-2,2-dimethyloctanoyl)-5,5-dimethyloxazolidin-2-one	1.506896156	0.011057102	up	1.460006606	0.017612402	down
MW0115484	xi-3-Methyl-3-cyclohexen-1-ol	1.372034019	0.040447034	up	1.460079668	0.005792369	down

MW0105658	Arg-Gly-Lys	1.500193882	0.002763541	up	1.393784635	0.004051767	down
MW0152872	Lys-Arg-Ser	1.548571123	0.014074191	up	1.17323694	0.039447118	down
MW0145467	Arg-Phe-Phe	1.534245271	0.015731819	up	1.305362321	0.02650305	down
MW0110365	Val-Pro-Lys	1.560470222	0.000254318	up	1.361354457	0.000305781	down
MW0152848	Lys-Ala-His	1.429687263	0.017899029	up	1.383932251	0.006145808	down
MW0052885	Farnesyl Thiosalicylic Acid Amide	1.686818803	0.008729187	up	1.315358161	0.00248435	down
MW0000320	Levorphanol	1.427019205	0.015778824	up	1.334785776	0.017650293	down
MW0143458	4S,5R-antillatoxin A	1.562670244	0.028204188	down	1.43308064	0.033602532	up
MW0116159	AM2201 N-(3-fluoropentyl) isomer	1.359211487	0.009743167	up	1.19424809	0.020744406	down
MW0125815	Nipecotic acid	1.638111344	0.023408144	up	1.277541578	0.030986746	down
MW0148315	Desalkyl verapamil D617	1.619201254	0.025242673	up	1.426845932	0.024503271	down
MW0108848	1-Methylpyrrolidine-2-carboxylic acid	1.432979889	0.038440123	up	1.374909619	0.04899323	down
MW0127264	2-Methyl-1-propenethiol	1.42348542	0.032969956	down	1.238559169	0.019734415	up

MW0109843	Thiorphan	1.624360591	0.017035081	up	1.236533773	0.007948812	down
MW0159895	(2R,3R)-2-Aminooctadecane-1,3-diol	1.301169315	0.042094813	down	1.295012095	0.001509377	up
MW0151418	Ile-Glu-Ile	1.401930504	0.011935168	up	1.471735435	0.004305912	down
MW0123391	Clomethiazole	1.62708845	0.022280647	up	1.34444029	0.007346057	down
MW0010841	(17alpha,23S)-Epoxy-28,29-dihydroxy-27-norlanost-8-ene-3,24-dione	1.680108582	0.003302495	up	1.468963141	0.007811299	down
MW0123318	Chromafenozide	1.629091846	0.024000546	up	1.474132022	0.029090446	down
MW0158455	Tyr-Glu-Gln	1.734536304	0.009382894	down	1.47128356	0.021274759	up
MW0152228	Leu-Arg-Leu	1.596116836	0.000457329	up	1.470495233	0.003262949	down
MW0157337	Talatisamine	1.366575448	0.045487649	up	1.365545961	0.004201698	down
MW0137885	Dehydrocarpaine II	1.699284496	3.81014E-05	up	1.438454307	5.36375E-06	down
MW0009319	P,P-Dioctyldiphenylamine	1.623985144	0.024368782	up	1.458118979	0.032894292	down
MW0110610	11(12)-EpETrE-EA	1.688639095	0.012548191	down	1.44807953	0.0266532 1	up
MW0009881	Tert-butyl 4-methylbenzoate	1.468219879	0.009329138	up	1.445445814	0.0016121 9	down

MW0014422	4alpha-Methylzymosterol	1.328164868	0.044303784	up	1.274083289	0.020530946	down
MW0141397	14,15-Epoxyemindole SB	1.726273081	0.023406589	up	1.354981582	0.027593552	down
MW0141736	1'H-5alpha-Androst-2-eno[3,2-b]indol-17beta-ol	1.598707404	0.023545308	up	1.46859403	0.027414106	down
MW0055808	PA(18:3(9Z,12Z,15Z)/18:1(9Z))	1.546861209	0.034911673	down	1.412432595	0.002205062	up
MW0169279	Huperzine-A	1.262861968	0.030207769	up	1.475727015	0.006764269	down
MW0152409	Leu-Leu-Val-Val-Ala	1.571883975	0.005303332	up	1.470909693	0.006831458	down
MW0169325	Isopentenyl-Adenine 35.0 eV	1.663590909	0.017769448	up	1.26570164	0.010763781	down
MW0011176	(3beta,17alpha,23S,24S)-17,23-Epoxy-3,24,29-trihydroxy-27-norlanost-8-en-15-one	1.337579613	0.049301894	up	1.453545918	0.006823342	down
MW0142592	3-HYDROXYDEOXODIHYDRODEOXYGEDUNIN	1.478075219	0.008955394	up	1.379669826	0.010942197	down
MW0145390	Arg-Ile-Glu-Asp	1.391498581	0.033947151	up	1.391140447	0.022556525	down
MW0152410	Leu-Leu-Val-Val-Tyr	1.672593635	0.015259298	up	1.256521075	0.018834737	down
MW0152912	Lys-Asp-Thr-Lys	1.573376046	0.014942997	up	1.443570088	0.023802946	down
MW0145733	Asn-Gln-Val-Lys	1.559355153	0.005791834	up	1.4017082	0.003026692	down

MW0152282	Leu-Gln-Arg-Arg	1.652592165	0.037833911	down	1.425320168	0.026063112	up
MW0145833	Asn-Lys-Leu-Arg	1.651609832	0.036925382	up	1.359196831	0.040346864	down
MW0153110	Lys-Phe-Val-Leu-Val	1.583374463	0.010505872	down	1.263485583	0.017106566	up
MW0141313	12(13)-EpOME-d	1.243450554	0.030552854	up	1.465653891	0.023576498	down
MW0148481	Dihomo--linolenoyl-EA	1.343802712	0.025725028	up	1.468299389	0.029028462	down
MEDL02209	Protopine	1.455488809	0.028768663	up	1.118360255	0.041992134	down
MW0126557	Simvastatin	1.638262846	0.004783196	up	1.379129115	0.002813063	down
MW0063692	Suberic acid	1.598091274	0.015491249	up	1.280181389	0.036005521	down
MEDN0685	Undecanedioic acid	1.534087305	0.030803863	up	1.413959494	0.031810393	down
MW0103073	Valeric acid	1.557203796	0.020115083	up	1.101232984	0.045176498	down
MW0063484	Sebacic acid	1.547507105	0.012272023	up	1.383546701	0.009430349	down
MW0015395	9S-HOTrE	1.699548324	0.030900215	down	1.42854486	0.033638312	up
MW0054697	Medicagenic acid	1.485540899	0.017090355	up	1.400301779	0.013792092	down

MEDN 0589	Cyclamic acid	1.724271 795	0.0175218 57	up	1.42179 9543	0.0108423 15	down
MW010 8213	Methionine	1.672085 925	0.0048481 82	up	1.26187 9601	0.0203360 94	down
MW010 6169	Citrulline	1.552432 826	0.0175618 25	up	1.16319 0745	0.0474658 91	down
MW013 3678	4-Hydroxycinnamic acid	1.395925 604	0.0187116 31	down	1.11131 727	0.0372761 92	up
ZINC83 83223	4-Hydroxycyclohexylcarboxylic acid	1.469867 822	0.0180514 63	up	1.47959 9782	5.90762E-05	down
ZINC15 62127	10-Hydroxy-2-decenoic acid	1.725674 379	0.0004333 56	up	1.47884 2342	0.0016479 37	down
ZINC73 032	3-(3,4,5-Trimethoxyphenyl)propanoic acid	1.687445 997	0.0068690 84	up	1.48226 8431	0.0094819 86	down
MW010 6090	Caffeic acid	1.611151 865	0.0023973 83	up	1.45932 6972	0.0089515 56	down
MW001 4823	5-oxoETE	1.721126 617	0.0379731 61	up	1.41523 0908	0.0384289 37	down
MEDT N00356	Proglumide	1.731050 576	0.0154227 55	up	1.42229 712	0.0091535 09	down
MEDN 1451	Resolvin E1	1.513676 985	0.0221891 34	up	1.43843 8488	0.0014354 99	down
MW005 3869	Heptanoic acid	1.560362 823	0.0217182	up	1.42714 0359	0.0169714 58	down
MEDN 1387	O-acetyl-L-serine	1.461449 792	0.0285005 47	up	1.13802 6817	0.0441461 66	down

MW0159289	Val-Met	1.703735614	0.011969817	up	1.437377978	0.013751806	down
MEDP2239	(S)-Acenocoumarol	1.26273053	0.021773111	up	1.387810851	0.000403332	down
MW0105910	Asn-Val	1.64106183	0.001016861	up	1.477877817	0.006309455	down
MEDL00344	Glycyl-L-tyrosine	1.56535776	0.012539753	up	1.458282991	0.02167617	down
MW0103675	Thymidine-5'-phosphate	1.43263886	0.016095315	up	1.477435601	0.003062276	down
MW0123579	Desthiobiotin	1.313415021	0.04260952	up	1.423457325	0.037678857	down
MEDP0327	N-γ-Acetyl-N-2-Formyl-5-Methoxykynurenamine	1.293750002	0.046346397	up	1.475318892	0.003186938	down
MW0107105	Glycyl-L-arginine	1.351039855	0.041632014	up	1.400584415	0.003797166	down
MEDL01839	8-Methyl-6-nonenoic acid	1.45520458	0.010756013	up	1.451586783	0.01898991	down
MW0108074	Lys-Ser	1.602158321	0.006403722	up	1.474052265	0.012217637	down
MW0014807	5-Methoxytryptamine	1.669125484	0.031485762	up	1.375273345	0.017995971	down
MW0010583	3,4-Bis[(3-hydroxyphenyl)methyl]oxolan-2-one	1.567991643	0.005154677	up	1.354256651	0.001312028	down
MW0017134	cis-11-Methyl-2-dodecenoic acid	1.625440174	0.004288213	up	1.40806127	0.003781585	down

MW0003089	2-Hydroxy-2-phenylpropanoic acid	1.625269586	0.041297027	down	1.451018626	0.000312971	up
MW0016270	Butyric acid	1.579318137	0.003085128	down	1.339261284	0.047986646	up
MW0104240	1-Aminocyclopropane-1-carboxylic acid	1.459417965	0.023318798	up	1.30326774	0.01337602	down
MW0112251	Butyl propenyl ketone	1.584168173	0.011141383	up	1.379481743	0.014136328	down
MW0109516	R-1 Methanandamide Phosphate	1.425687329	0.029864579	up	1.446089159	0.0047338	down
MW0014719	5beta-Cholanic acid	1.695576359	0.01698575	up	1.457426067	0.018652162	down
MW0006597	Citalopram	1.69599521	0.016904986	down	1.348153515	0.026163539	up
MW0014127	3-Hydroxytetradecanedioic acid	1.495269109	0.016172671	up	1.151768947	0.043708634	down
MW0007560	Mebhydrolin	1.673449632	5.61055E-05	up	1.475137108	0.00019756	down
MW0169673	Picrotin	1.637268822	0.004386636	up	1.482432012	0.006736772	down
MW0156513	Samandarone	1.346617463	0.049651575	up	1.301320353	0.031137692	down
MW0158343	Tylophorine	1.661433924	7.45736E-05	up	1.339636055	0.005627988	down
MW0126375	Quindoxin	1.575135778	0.010325007	down	1.188986133	0.047959082	up

MW0123630	diethyl 2,6-dimethyl-4-oxo-4h-pyran-3,5-dicarboxylate	1.666706169	0.00795499	up	1.477820688	0.010423252	down
MW0014813	5-Methylhexanoic acid	1.736650885	0.017134427	up	1.457711807	0.015887728	down
MW0014190	3-Methylglutarylcarnitine	1.377577553	0.002632832	up	1.432746112	0.008504007	down
MW0005467	Hesperetin	1.449834069	0.010211338	up	1.414515931	0.009797853	down
MW0014510	4-Hydroxyvalproic acid	1.599565778	0.012296775	up	1.440087638	0.021019533	down
MW0011380	(4S,8R)-8,9-Dihydroxy-p-menth-1(6)-en-2-one	1.617291819	0.037791459	up	1.29193422	0.024036838	down
MW0017100	Chrysanthemic acid	1.568697924	0.004066036	up	1.442255113	0.005933898	down
MW0147914	Cycleanine	1.460912611	0.012823161	up	1.457896359	0.029938342	down
MW0062252	Prostaglandin F2beta	1.393745201	0.047850292	down	1.360561084	0.005333336	up
FDATN00477	Moclobemide	1.515514554	0.007556724	up	1.443709712	0.016036542	down
MW0168446	12-Hydroxy-16-heptadecynoic acid	1.308268366	0.031751594	up	1.451679067	0.028901118	down
MW0011427	(6alpha,22E)-6-Hydroxy-4,7,22-ergostatrien-3-one	1.430024006	0.040987647	up	1.411336389	0.003053316	down
MW0122981	Austidiol	1.636251201	0.004019354	up	1.474262295	0.009862135	down

MW0108426	N-(3-Indolylacetyl)-L-isoleucine	1.51542981	0.010448876	up	1.404998733	0.018535385	down
MW0012045	11,12,14-Trihydroxy-7-methoxy-8,11,13-abietatrien-20,6-olide	1.698051355	0.000376943	up	1.451443812	0.00147282	down
MW0115374	Tomelukast	1.318143992	0.015032756	up	1.460232681	0.017872974	down
MW0006200	MS-Ppoh	1.479088423	0.007796392	up	1.444807064	0.013575246	down
MW0000170	N-Methylactinodaphnine	1.48584529	0.024618501	up	1.463247242	0.002861533	down
FDATN00935	Sulfisomidine	1.713967522	0.018697857	up	1.457079541	0.016403907	down
MW0005841	6beta-Naltrexol	1.683104258	0.000877911	up	1.233935535	0.043864496	down
MW0103535	2',3'-Dideoxyinosine	1.537250932	0.022217941	up	1.450878396	0.023826755	down
MW0128365	(R)-Equol	1.640295621	0.000758894	up	1.282686482	0.00080137	down
MW0054639	Macrocarpal C	1.474074384	0.011147314	up	1.374814977	0.026560251	down
MW0009370	Pevonedistat	1.504327964	0.035428375	up	1.300230464	0.037184492	down
MW0108431	N-(3-Oxododecanoyl)homoserine lactone	1.303636774	0.015308121	up	1.35321397	0.020167279	down
MW0105445	Agomelatine	1.481408218	0.012567736	up	1.386572302	0.004869908	down

MW0109269	Picrotoxinine	1.742842423	0.010833056	up	1.482215756	0.010833056	down
MW0007814	Mibefradil	1.533687515	0.016378189	up	1.407085108	0.025415888	down
MW0153950	Monocerin	1.346157022	0.028869634	up	1.201072413	0.008968202	down
MW0052422	Dioscin	1.644825813	0.0197112	down	1.432572432	0.042123244	up
FDATN00851	Tacrolimus	1.538357125	0.012304054	down	1.350594755	0.014482868	up
MW0123716	Dinaciclib	1.171534713	0.032556382	up	1.420384872	0.000454673	down
MW0147048	Candimine	1.680465268	0.010429391	up	1.340363521	0.007073258	down
MW0009985	Trimethoprim	1.601401329	0.001837715	up	1.430613817	0.009546368	down
MW0062149	Prednisolone	1.469973218	0.005196215	up	1.444173918	0.000688429	down
MW0157976	trans-1,4-Dichloro-2-butene	1.607895007	0.005103683	up	1.349248173	0.0260446 3	down
MW0052991	beta-D-Glucopyranoside, (3beta)-solanid-5-en-3-yl	1.555148005	0.0101314 6	up	1.404638305	0.010732591	down
MW0125749	Nefazodone	1.337461825	0.015675177	up	1.23607757	0.004870546	down
MW0125812	Nimodipine	1.71683817	0.000157139	up	1.461297982	0.000757203	down

MW0115194	(2S)-2-(hydroxymethyl)-2,5,7-trimethyl-6-[2-[(2R,3R,4S,5S,6R)-3,4,5-trihydroxy-6-(hydroxymethyl)oxan-2-yl]oxyethyl]-3H-inden-1-one	1.473230771	0.046239004	up	1.41406658	0.0462772 3	down
ZINC77999	4-Methoxycinnamic acid	1.286635254	0.014941091	up	1.406828408	0.001030054	down
MW0007203	Flutriafol	1.559787567	0.006848365	down	1.224554744	0.033757109	up
MW0120159	Bucharaine	1.375342214	0.034423723	up	1.289967674	0.039459678	down
MW0014105	3-Hydroxynonyl acetate	1.693871292	0.028095811	up	1.318304508	0.023483351	down
MW0112810	3-Furanmethanol glucoside	1.388590886	0.0234626 1	up	1.250236849	0.018370547	down
MW0102929	Tricyclene	1.693247173	0.004531721	up	1.270893503	0.020676838	down
MW0109534	Ropivacaine	1.534753438	0.041594982	up	1.301362838	0.045657927	down
MW0053485	Geranyl acetate	1.593159271	0.010073578	up	1.441686688	0.013863205	down
MW0120164	2-Hexyl-4-acetoxytetrahydrofuran	1.571872518	0.004863857	up	1.481249617	3.85136E-05	down
MW0052493	Drospirenone	1.416716764	0.047598517	up	1.415592768	0.003090248	down
MW0109212	Phenylalanylserine	1.390020174	0.023701926	up	1.447016287	0.000891127	down
MW0105831	Asn-Pro	1.56107333	0.010859726	down	1.424092641	0.006490709	up

MW0159789	HSO3-	1.47258997	0.042480129	up	1.441335562	0.018311315	down
MW0142358	(4R,6R)-6-[2-[(1S,2S,8aR)-2-methyl-1,2,6,7,8,8a-hexahydronaphthalen-1-yl]ethyl]-4-hydroxyoxan-2-one	1.418048194	0.040003351	up	1.155876713	0.037196756	down
MW0016190	(-)-Bilobalide from ginkgo leaves	1.493536239	0.011260988	up	1.447958123	0.011892925	down
MW0054417	5,12-Dihydroxyicosa-6,8,10-trienoic acid	1.656382617	0.005154246	down	1.259425704	0.035315695	up
MW0141608	17-keto-7(Z),10(Z),13(Z),15(E),19(Z)-Docosapentaenoic Acid	1.521004204	0.01666379	up	1.212202456	0.015612201	down
MW0128359	(R)-3',7-Dihydroxy-2',4'-dimethoxyisoflavan	1.523452926	0.004020361	down	1.217357679	0.014363035	up
MW0142824	3beta,21-Dihydroxy-pregna-5,7,9(11)-trien-20-one diacetate	1.511667347	0.001844615	up	1.468104567	0.00814184	down
MEDN0784	9-OxoODE	1.68853708	0.028942749	down	1.443682535	0.035166263	up
MW0169366	D-Kynurenine	1.560114815	0.018135175	up	1.367339232	0.020460812	down
MW0122525	7-Methyluric acid	1.638334106	0.027501753	down	1.403260494	0.01863755	up
MW0111044	3-Guanidinopropionic acid	1.525927336	0.002778775	up	1.467840379	0.006992594	down
MW0009360	Pentoxifylline	1.607159235	0.048032538	up	1.350153478	0.033431175	down
MW0140257	(25R)-3-ketocholest-4-en-26-al	1.345396837	0.029957708	up	1.450693957	0.012048696	down

MW0055804	PA(18:3(9Z,12Z,15Z)/16:0)	1.41652577	0.043331536	up	1.447533822	0.02473353	down
MW0055805	PA(18:3(9Z,12Z,15Z)/16:1(9Z))	1.640891135	0.000839068	up	1.414988957	0.000635803	down
MW0013641	2-Polyprenyl-3-methyl-5-hydroxy-6-methoxy-1,4-benzoquinone	1.412992231	0.015571421	up	1.219290853	0.032820384	down
MW0009644	Didemethylcitalopram	1.35282467	0.037854555	up	1.204509562	0.049641747	down
MW0105268	2-n-Propyl-4-oxopentanoic acid	1.39597893	0.027126485	up	1.472017537	0.008578588	down
MW0130859	2-Hydroxyibuprofen	1.580723549	0.012451449	up	1.378721391	0.007444376	down
MW0140325	(2S,3S)-2-hydroxytridecane-1,2,3-tricarboxylic acid	1.670744986	0.031351936	up	1.304650391	0.024406593	down
MW0014677	5-Acetamidovalerate	1.576855143	0.012022052	up	1.223811196	0.025153467	down
MW0115431	Triptolide analog	1.673022256	0.03272399	up	1.45472513	0.030966736	down
MW0054091	Isohumulinone A	1.591939591	0.01317732	up	1.464316587	0.007548008	down
MW0143591	5-cis-15(R)-Iloprost	1.484521985	0.020544616	up	1.276286795	0.035839833	down
MW0006081	Ascochitine	1.401387638	0.008008317	up	1.471361212	2.03812E-05	down
MW0165281	Cyclooctatin	1.666314442	0.03149982	up	1.307290473	0.023087796	down

MW0012793	1-Eicosanoyl-glycero-3-phosphate	1.607795856	0.031729818	down	1.419604139	0.003598341	up
MW0104208	10-Hydroxydecanoic acid	1.57911688	0.005065951	up	1.471973461	0.015009767	down
FDATN01294	Besifovir	1.263194205	0.047321357	up	1.432646823	0.044480856	down
MW0154500	Nigakilactone A	1.405183602	0.045245793	up	1.442691021	0.000668932	down
MW0052599	Eremopetasitenin C1	1.519486595	0.032990047	up	1.366215876	0.032821658	down
MW0014915	6-(2-Benzyl-3-oxobutoxy)-3,4,5-trihydroxyoxane-2-carboxylic acid	1.515569882	0.002506067	up	1.479725305	0.006665942	down
MW0004656	4-amino-N-(4-tert-butylphenyl)benzenesulfonamide	1.581649122	0.029222631	up	1.439089182	0.040811935	down
MW0059312	PE-NMe(15:0/22:0)	1.593443369	0.015417599	down	1.374487341	0.014877091	up
MW0107353	His-Thr	1.651660062	0.00782308	up	1.444399075	0.010910668	down
MW0132908	3-[3,4-dihydroxy-5-(3-methylbut-2-en-1-yl)phenyl]-5,7-dihydroxy-8-(3-methylbut-2-en-1-yl)-3,4-dihydro-2H-1-benzopyran-4-one	1.506451289	0.002279946	up	1.425120197	0.002532493	down
MW0012054	11,12-Dimethoxy-8,11,13-abietatrien-20,7-olide	1.63554335	0.005697092	up	1.328842697	0.004915036	down
MW0145233	Arctiopicrin	1.480454663	0.013603853	up	1.409097095	0.018214974	down
MW0055827	PA(18:3(9Z,12Z,15Z)/22:6(4Z,7Z,10Z,13Z,16Z,19Z))	1.739689666	0.017141116	up	1.478718075	0.017141116	down

FDATN00255	Ixazomib	1.241636376	0.04162742	up	1.413964745	0.038029321	down
MW0056577	[(2R)-1-(11-methyldodecanoyloxy)-3-phosphonooxypropan-2-yl] 13-methyltetradecanoate	1.410528604	0.030083587	down	1.280290768	0.049516116	up
MW0006489	Carbaryl	1.694341691	0.019844867	up	1.346380645	0.011380064	down
MW0110310	L-Tyrosyl-L-Threonine	1.406794844	0.009861077	up	1.476239234	0.00561133	down
MW0145984	Asparaginyl-Lysine	1.599896875	0.009409718	up	1.463961994	0.01612991	down
MW0145980	Asparagylisoleucine	1.645338988	0.007648603	up	1.418597243	0.00367952	down
MW0145995	Asn-Trp	1.375846534	0.014239602	up	1.430181508	0.009505881	down
MW0145670	Asn-Arg-Gln-Lys	1.589328195	0.01031384	down	1.32028023	0.025334683	up
MW0144245	Acetoxy-6-gingerol	1.411301167	0.03971282	up	1.253818174	0.041994135	down
MW0144246	Acetoxy-8-gingerol	1.409837403	0.020077391	up	1.199585915	0.021659307	down
MW0141664	19(R)-hydroxy-PGB2	1.410381509	0.003565946	up	1.29238263	0.005015316	down
MW0011173	(3beta,17alpha,23S)-17,23-Epoxy-3,28,29-trihydroxy-27-norlanost-8-en-24-one	1.433846881	0.047607074	up	1.436412678	0.035620103	down
MW0153100	Lys-Phe-Leu-Glu	1.591789907	0.02202917	down	1.334896267	0.032035945	up

MW0145690	Asn-Asn-Leu-Asn-Val	1.602665834	0.039665312	up	1.442140372	0.046968836	down
MW0139559	Resveratrol	1.562745047	0.010200772	up	1.262569736	0.001234352	down
MW0145518	Arg-Thr-Ile-Glu	1.714647254	0.01605934	down	1.437505334	0.042975187	up
MW0155344	Phe-Thr-Arg-Lys	1.626669982	0.015246089	up	1.407526092	0.004863151	down
MW0144650	Ala-Leu-Val-Arg	1.355648958	0.048270775	up	1.47133845	0.01618105	down
MW0151553	Ile-Phe-Val-Lys	1.416093071	0.026236013	up	1.400156088	0.003216164	down
MW0106327	Cysteinyl-Methionine	1.49748822	0.00874121	up	1.466503357	0.018897513	down
MW0141365	13,14-dihydro-16,16-difluoro PGJ2	1.507681584	0.024505147	up	1.466243987	0.038479866	down
MW0143330	4-hydroxy Nonenal Mercapturic Acid-d3	1.619510068	0.00483187	up	1.42209091	0.00029823	down
MW0144205	Abu-Ile-OH	1.720907744	0.011094198	down	1.421299123	0.030178432	up
